# A GTP synthase ribozyme with increased GTP turnover

**DOI:** 10.1073/pnas.2520997123

**Published:** 2026-06-09

**Authors:** Xu Han, Zoe J. Pepper, Joshua T. Arriola, Ulrich F. Müller

**Affiliations:** ^a^ Department of Chemistry and Biochemistry, University of California San Diego, La Jolla, CA 92093

**Keywords:** ribozyme, NTP, in vitro selection, emulsion, origin of life

## Abstract

Before the invention of encoded protein translation, early stages of life likely relied on catalytic RNAs (ribozymes). To test how such a system could have functioned, researchers have developed ribozymes that could have provided central functions. The central function of self-replication would have required templated RNA polymerization of nucleotides, which is energetically driven in today’s life forms by the use of nucleoside 5′-triphosphates (NTPs). We previously showed that ribozymes can catalyze the formation of guanosine 5′-triphosphate (GTP) from guanosine and the prebiotically plausible polyphosphorylation reagent cyclic trimetaphosphate (cTmp) by generating a guanosine triphosphorylation ribozyme (GTR) using an in vitro selection in emulsion. This ribozyme (GTR1) had a catalytic rate enhancement of about 18,000-fold but a turnover of only about 1.7. Here, we improved this ribozyme by emulsion selection from a doped library of GTR1 that was metabolically coupled to a polymerase ribozyme. High-throughput sequencing and biochemical analysis identified the most efficient variant of GTR1 with 19 mutations, which increased the GTP turnover number to ~13. Biochemical analysis of this GTR1e revealed biphasic reaction kinetics with an apparent overall K_M_^APP^ around 11 mM for cTmp. When coupled to an RNA polymerase ribozyme, up to five guanosines were incorporated into an RNA polymer, which represents an important step toward modeling an RNA-based life form in the lab.

## Significance

An early stage of life must have used catalytic RNAs (ribozymes) to self-replicate in a prebiotic environment. Self-replication would have required RNA polymerization from chemically activated nucleotides, which exist in today’s biology as nucleoside 5′-triphosphates (NTPs). We previously developed a ribozyme that is able to synthesize guanosine 5′-triphosphate (GTP) by metabolic coupling between two ribozymes in emulsion droplets. Here, we improved the GTP turnover of this ribozyme by selection in emulsion from a library of ribozyme variants. The resulting ribozyme had a GTP turnover of about 13, and a coupled reaction with an RNA polymerase ribozyme led to the incorporation of five guanosines into an RNA polymer. This represents an important step toward recapitulating an early RNA-dominated stage of life in the lab.

Early life forms likely relied on RNA molecules to support self-replication and a metabolism (1–4). This idea is supported by the ribosome being a highly evolved catalytic RNA (ribozyme) (5), by many cofactors being nucleotides or their derivatives (6), and by the nucleotide cofactor pairs ATP/ADP and NAD^+^/NADH being the most centrally connected metabolic couples next to protons and water (7, 8). At this RNA dominated stage, catalytic RNAs (ribozymes) must have mediated self-replication and a metabolism. Unfortunately, macromolecules from that stage cannot be recovered from sediments of that time because they have long degraded over the past 3.5 billion years (9, 10). To test how such an RNA system could have functioned, researchers have developed ribozymes that would have had central functions in that system. The two central functions are self-replication, and a metabolism that supports this self-replication. These two functions are tightly linked because the RNA system would only be able to sustain itself when the catalysis of metabolic reactions provides a sufficient amount of activated monomers for self-replication. The development of new ribozymes is possible by a method called in vitro selection, where large libraries with randomized RNA sequences are successively enriched for RNAs with the desired function (11–13). A ribozyme with the central activity of template-dependent RNA polymerization was developed by in vitro selection (14) and improved in efficiency and fidelity toward the goal of a self-replicating system (15–17). The substrates of these polymerase ribozymes are nucleotides or oligonucleotides with 5′-triphosphates as chemically activated 5′-phosphates. The central position of nucleoside 5′-triphosphates (NTPs) for RNA synthesis in all known organisms (8, 18) suggests that late RNA-based life forms also used NTPs as central energy metabolite. To test these ideas in the lab, a critical proof of principle would be to metabolically couple multiple turnover ribozymes that synthesize such NTPs with ribozymes that use these NTPs for RNA polymerization.

We previously selected a ribozyme that generates free GTP from guanosine and cyclic trimetaphosphate (cTmp) (19). Different routes for the prebiotic production of cTmp exist, including their production in volcanic environments, the erosion and oxidation of meteoritic Schreibersite minerals, the percolation of phosphate-rich water through iron-rich sediments, and lightening-induced conversions of phosphate minerals (20–23). The direct in vitro selection of a ribozyme generating a freely diffusing product (in this case GTP) was made possible by performing the selection step under experimentally carefully controlled conditions in emulsion, where GTP synthesis was metabolically coupled with a second ribozyme that covalently linked the reaction product with successful catalysts (19). This emulsion required a large number of droplets (in this case 10^16^) and a narrow droplet size distribution [in this case at a 150 nm diameter (24)] so that the majority of the 5 × 10^15^ library RNAs occurred as the only RNA library molecule within their droplets. The emulsion droplets contained a specially optimized polymerase ribozyme (25) to tag successful catalysts at their 3′-terminus. During the selection, 6-thio guanosine (6sG) was used instead of guanosine so that successful catalysts were tagged with a sulfur modification and could be isolated via their specific and strong affinity to immobilized mercury (26–28). This selection scheme generated a ribozyme that catalyzed the formation of GTP from guanosine and cTmp with about 18,000-fold rate enhancement over the uncatalyzed reaction (19, 29, 30). Its low GTP turnover of only 1.7 (19) was likely the result of the selection conditions, which were designed to identify even weakly active GTP synthase ribozymes: At the used emulsion droplet diameter of 150 nm, the production of a single molecule of 6sGTP inside a droplet corresponds to a concentration of 0.94 μM. This was expected to thio-label a single library molecule within the same droplet with more than 90% efficiency during the selection step (24, 25). In addition, the successive decrease of substrate concentrations over the selection rounds may have favored library molecules that bind tightly to 6sG and cTmp substrates, which may inhibit turnover by slow release of the 6sGTP product.

Here, we show the identification of a GTP synthase ribozyme with increased GTP turnover, using metabolic coupling with an RNA polymerase ribozyme in emulsion. The selection pressure was fine-tuned by adjusting droplet size, substrate concentration, and polymerase ribozyme activity. After high-throughput sequencing (HTS) analysis and biochemical screening, a GTP synthase ribozyme with a GTP turnover of ~13 was identified (GTR1e). The ribozyme had an apparent, overall K_M_^APP^ for cTmp of ~11 mM, and an apparent K_M_^APP^ for guanosine of about 1 mM. When the GTP synthesis of GTR1e was metabolically coupled to ribozyme-catalyzed RNA polymerization, up to five guanosines were incorporated into the RNA polymer.

## Results

**Fig. 1.**
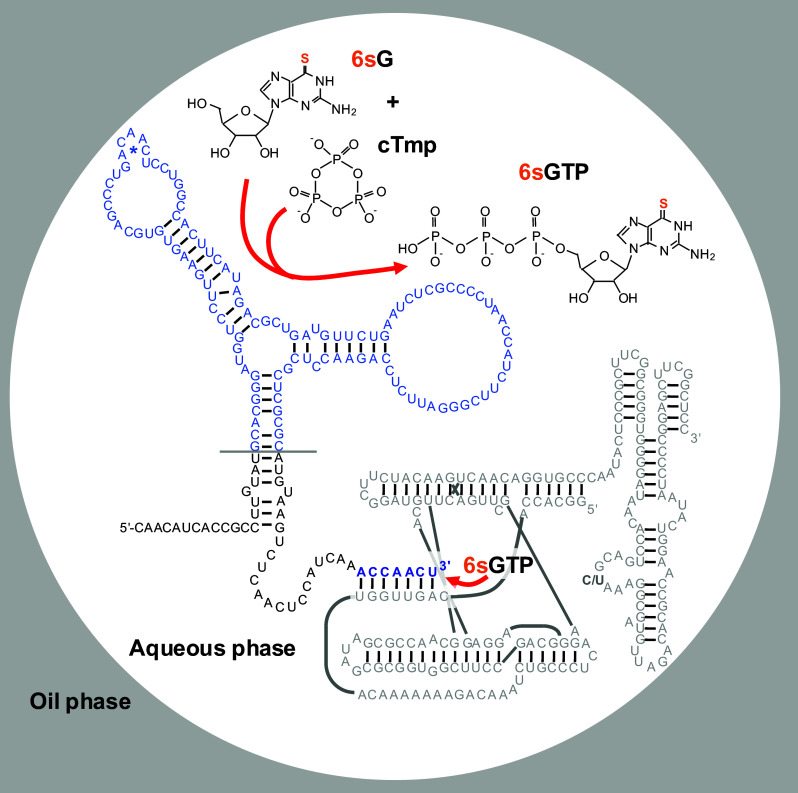
Schematic of the selection step in the doped *in emulsio* selection. The oil phase is depicted as a gray background and the aqueous droplet as white circle. The droplet contains two RNA molecules: The doped library of GTR1 variants (*Left*) contained a partially randomized sequence (light blue) and its 3′-terminus (dark blue) was base paired to the polymerase ribozyme (*Right*; gray). The GTP synthase library was challenged to catalyze the reaction between 6sG and cTmp to 6sGTP (top red arrow). The chemical structures of 6sG, cTmp, and 6sGTP are shown. Reaction conditions were 1 mM guanosine, 50 mM Na_3_cTmp, 150 mM MgCl_2_, 200 mM KCl, 50 mM Tris/HCl pH 8.0 at 22 °C for 1 h. The 6sGTP was then used by the polymerase ribozyme to tag the 3′-terminus of the GTR library (dark blue and bottom red arrow). The GTR1 library was truncated internally (*) to reduce the total length of the partially randomized region to 116 nucleotides. The library 3′-terminus (dark blue) was processed by an M1 RNase P ribozyme for homogeneous 3′-ends (*SI Appendix*, Fig. S3). Two polymerase ribozyme variants were used, one of them with sevenfold lower activity where a U in the NTP binding loop at position 156 was replaced by a C (bold dark gray). The diameter of the aqueous droplets was adjusted between ~150 nm and ~400 nm by modifications to the emulsification procedure.

To identify variants of the GTP synthase ribozyme GTR1 with an increased GTP turnover, a doped selection was performed under conditions that were modified from the emulsion selection conducted previously (Fig. 1) (19). As before, an RNA library was compartmentalized in emulsion droplets together with a polymerase ribozyme, 6sG, and cTmp. Sequences that catalyzed the reaction between 6sG and cTmp were tagged with 6-thio GTP (6sGTP) at their 3′-terminus using the polymerase ribozyme. The emulsification reduced the diffusion of produced 6sGTP molecules from active to inactive library molecules. After recovering the RNA library from the emulsion, thio-tagged RNA library molecules were isolated on mercury containing APM (aminophenyl mercury) polyacrylamide gels (*SI Appendix*, Fig. S1) (26–28) and amplified by reverse transcription, PCR, and transcription to complete one selection round. The library was truncated at an internal stem-loop to help cover sequence variants (*SI Appendix*, Fig. S2). The 3′-terminus of the ribozyme library was processed by the M1 RNase P ribozyme to help tagging (*SI Appendix*, Fig. S3), and the reaction time in emulsion was optimized to 1 h to favor GTP synthases with increased turnover (*SI Appendix*, Fig. S1). To help select library sequences mediating higher turnover, three aspects were modified from the previous procedure (*SI Appendix*). First, the concentrations of the substrates were kept high to reduce the need for tight substrate binding and the possible consequence of product inhibition. Second, the emulsion droplet size was increased from 150 nm diameter to about 400 nm diameter (*SI Appendix*, Fig. S1). Third, a polymerase ribozyme was used for metabolic coupling that had sevenfold lower activity and therefore required more 6sGTP for efficient coupling (25). While the substrate concentrations were kept high in all three lines of the selection, the droplet diameter and the polymerase ribozyme activity were varied in three lines of the selection: Line LL employed a large droplet diameter and low activity polymerase ribozyme, line LH employed a large droplet diameter and a highly active polymerase ribozyme, and line SH employed a small droplet diameter and a highly active polymerase ribozyme. The first round of the selection covered an effective complexity of 1.2 × 10^13^ sequences (*Materials and Methods*). With a doping ratio of 6% in the 116 partially randomized positions of the library (94% parent sequence and 2% of each mutation), we expected this complexity to cover all single, double, triple, quadruple, and quintuple mutations in this region (*SI Appendix*, Fig. S4).

After three selection rounds (selection line SH) or 9 selection rounds (selection lines LL and LH), the selected populations of sequences were subjected to HTS analysis, and several mutations and covarying sequence motifs were found highly enriched (*SI Appendix*, Fig. S6). Fifteen highly enriched sequences were tested biochemically for their GTP synthesis (*SI Appendix*, Fig. S7). The strongest signal for GTP synthesis was generated by GTR variant SH4, the fourth-highly enriched sequence in selection line SH. To test whether the activity of SH4 could increase further, ten enriched mutations and six covariations were introduced into the context of SH4. The most active sequence variant of SH4 contained the U64C mutation, was named GTR1e (“evolved GTR1”), and was characterized in more depth.

**Fig. 2.**
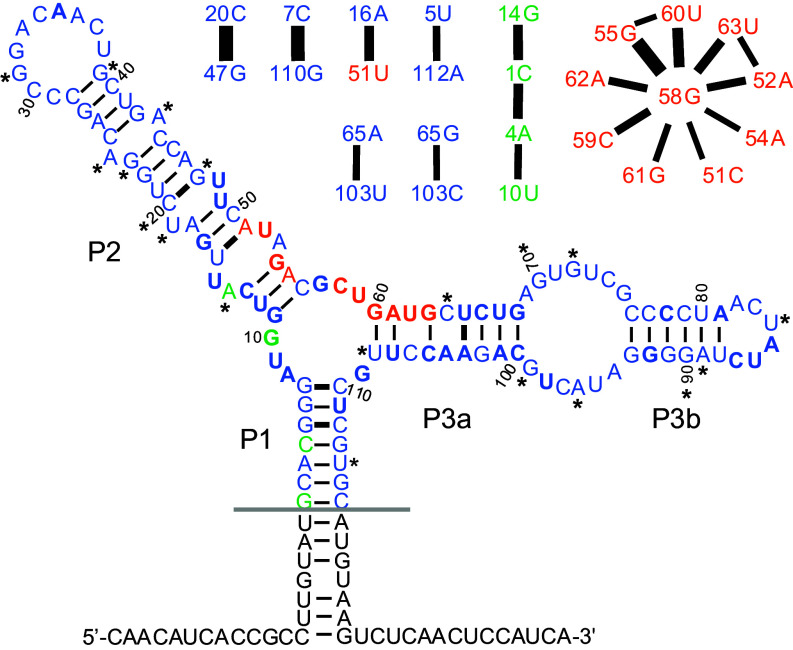
Secondary structure model of GTR1e with results from the HTS analysis. Colored nucleotides indicate positions in the doped region. Asterisks show the 19 mutations in GTR1e relative to the parent sequence. Positions shown in bold were conserved among the top 20 sequences of the three lines of selection (the top 10 sequences from each line, which resulted in a total of 20 due to overlaps between lines). The schematics on top describe the epistatic interactions formed by the 20 strongest base covariations in selection line SH. The thickness of the black lines corresponds to the strength of the epistatic interactions (*Materials and Methods* and *SI Appendix*). Thick black lines in the secondary structure indicate base pairing interactions that were among the 20 strongest epistatic interactions. Positions shown in orange formed an epistatic interaction network, and positions shown in green formed a separate epistatic interaction network. Weaker epistatic interactions confirmed the stems P1, P2, P3a, P3b (*SI Appendix*, Fig. S8). The colors of the positions in the epistatic interactions correspond to the same colors in the secondary structure. The nucleotide numbering denotes the position in the doped sequence. The horizontal gray line shows the beginning and end of the doped sequence.

The results from HTS analysis were projected onto an improved secondary structure model (Fig. 2). This secondary structure was based on the experimentally determined secondary structure for the parent ribozyme (19), modified to the sequence of GTR1e, and adjusted with the epistatic map of double mutations, which identified base pairs (*SI Appendix*, Fig. S8). Importantly, the overall structure (Fig. 2) was confirmed as a three-way helical junction of the P1 stem, P2 stem, and P3a stem. The new P3b stem was stabilized by two mutations in SH4 at positions 89 and 90. In addition, two networks of interacting nucleotides were identified (orange and green in Fig. 2; supporting evidence in *SI Appendix*, Figs. S9 and S10). These networks include several unpaired bases and noncanonical base pairs in a highly conserved region, suggesting that they represent the catalytic core of the ribozyme.

**Fig. 3.**
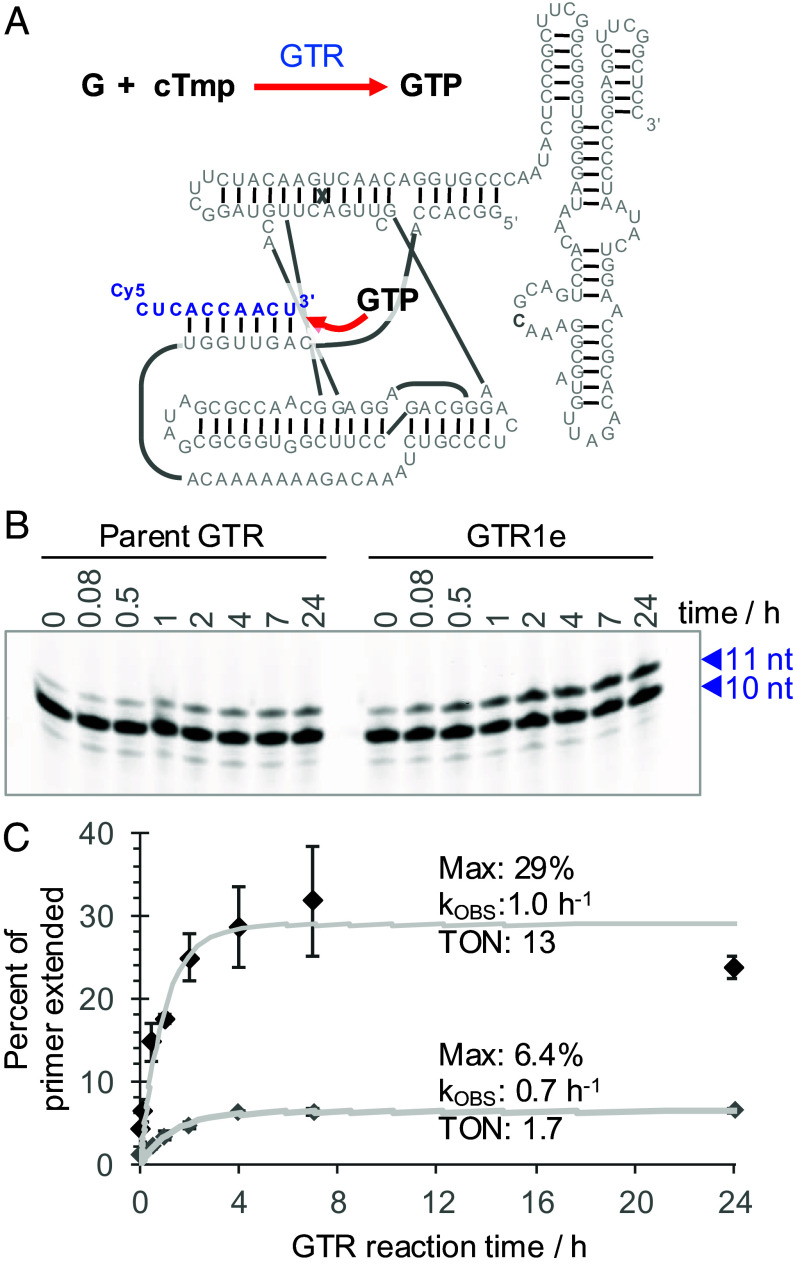
Kinetic analysis of the guanosine triphosphoprylation ribozyme GTR1e in comparison to the parent GTR. (*A*) Schematic of the assay that measures GTP synthesis (upper red arrow) by the GTP triphosphorylation ribozyme (GTR; light blue) from guanosine (G) and cTmp. The produced GTP is used as substrate (lower red arrow) by a polymerase ribozyme (gray) to extend a primer with a Cy5 label at its 5′-terminus (dark blue). (*B*) Image of Cy5-labeled reaction products separated by denaturing polyacrylamide gels. Each GTR was incubated for the indicated time with guanosine and cTmp before the reaction mixture was diluted 360-fold into a reaction containing the Cy5-labeled primer and the polymerase ribozyme that extended the primer using GTP. The lengths of the unextended primer (10 nt) and the extended primer (11 nt) are indicated. (*C*) Graph showing the signals in the GTP synthesis assay as function of the incubation time of the GTR with guanosine and cTmp. Black symbols show the data from GTR1e, gray symbols show data from the parent ribozyme. Error bars are SD from triplicate experiments, with error bars smaller than the symbols if not fully visible. Gray curves are single-exponential curve fits to the averages, with the fitted kinetic parameters given in the insert. The GTP TON was calculated from the maximum (Max) of the single-exponential curve fits, the dilution of the GTR into the primer extension reaction, and the concentration of GTR as well as the concentration of Cy5-labeled primers in both reactions (*Materials and Methods*).

To measure the increase in GTP turnover, the guanosine triphosphorylation kinetics were determined for the new ribozyme GTR1e and the parent ribozyme in a kinetic assay (Fig. 3). The GTP turnover number (TON) was 10 to 13 for GTR1e, about sevenfold higher than the parent GTR. The GTP turnover was 1.7 for the parent ribozyme, consistent with the previous study (19).The value of 13 for GTR1e results from the maximum amplitude of the single-exponential fit to the data, and it was 14 when the average signal after 7 h reaction (32% primer extended) was used (Fig. 3*C*). The average signal after 20 h correlated with a TON of 10.2. The observed reaction rates were similar for the two ribozymes with 0.7 h^−1^ for the parent GTR1 and 1.0 h^−1^ for GTR1e. Given that 36 nucleotides of the ribozyme’s 156 nucleotides are guanosines, the 13 GTP molecules produced by each GTR1e ribozyme represent about a third of the GTP that would be necessary for the ribozyme’s own synthesis.

**Fig. 4.**
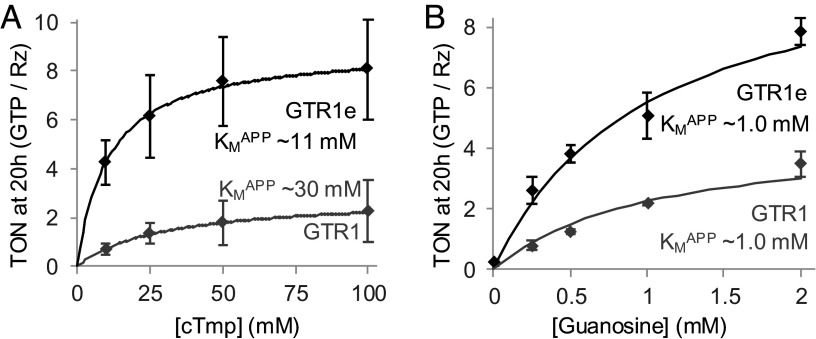
Dependence of ribozyme catalyzed GTP synthesis on substrate concentrations. (*A*) Dependence of GTP synthesis on the cTmp concentration, at a guanosine concentration of 2 mM. Shown is a plot of the total amount of GTP produced per ribozyme after 20 h reaction time for GTR1e (black) and GTR1 (gray). The lines are fit to the Michaelis–Menten equation, with the resulting constants given. The K_M_^APP^ is not a true K_M_ because it was derived from final product amounts and not reaction velocities. (*B*) Dependence of GTP synthesis on the guanosine concentration, at a cTmp concentration of 200 mM. Shown is a plot of the total amount of GTP produced per ribozyme after 20-h reaction time for GTR1e (black) and GTR1 (gray). The lines are fit to the Michaelis–Menten equation, with the resulting constants given. The K_M_^APP^ is not a true K_M_ because it was derived from final product amounts and not reaction velocities. Error bars are SD of triplicate experiments.

The dependence of the GTP TON on the substrate concentrations was described well by the Michaelis–Menten equation (Fig. 4). Variation of the cTmp concentration suggested an apparent K_M_^APP^ (cTmp) of 11 mM, while changes in the guanosine concentration revealed an apparent K_M_^APP^ (guanosine) of 1.0 mM. The K_M_^APP^ (cTmp) was improved almost threefold over the 30 mM K_M_^APP^ (cTmp) for the parent ribozyme, while the K_M_^APP^(guanosine) remained constant between the parent ribozyme and GTR1e. An initial kinetic analysis suggested a biphasic behavior (*SI Appendix*, Figs. S11 and S12). Together, this analysis shows that GTR1e has improved interactions with cTmp and a biphasic kinetic behavior.

**Fig. 5.**
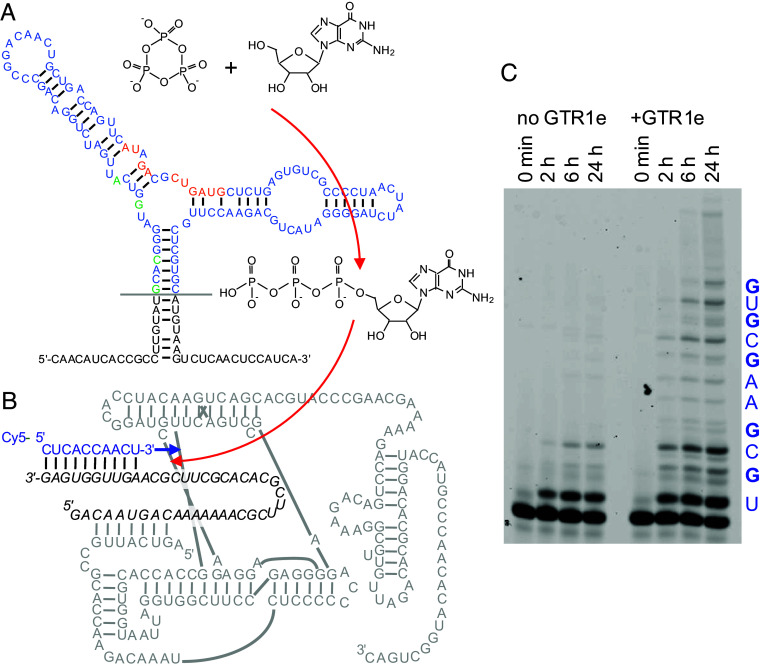
Ribozyme-mediated RNA polymerization based on metabolic coupling to GTR1e—generated GTP. The schematic of the two coupled reactions shows (*A*) the secondary structure of GTR1e, with the same color coding as in Fig. 2, and (*B*) the secondary structure of the polymerase ribozyme 71-89 on bottom, base paired to a template (italics) and a primer (dark blue) with a 5′-terminal Cy5 label. The dark blue arrow indicates the direction of primer extension with provided ATP, CTP, and UTP each at 0.2 mM concentration. (*C*) Scan of a denaturing 7 M urea 20% polyacrylamide gel separation of polymerization products from a 30 uL polymerization reaction that was mixed with 10 μL of a 20-h GTR1e GTP synthase reaction (*Right*) or 10 μL of a buffer containing the same components except GTR1e. The time points for the polymerization are indicated. Labels on the right show the templated nucleotide.

To test whether the improved GTP turnover of GTR1e was sufficient for the incorporation of multiple guanosines into an RNA polymer, a coupled reaction was set up between GTP synthesis and RNA polymerization (Fig. 5). The polymerase ribozyme 71-89 (16) was paired to a primer/template duplex and incubated with 0.2 mM ATP, CTP, and UTP. This means that the ribozyme could extend the primer on template nucleotides U, G, and A but not C. When one volume of GTP synthesis reaction was mixed with three volumes of a mixture containing polymerase ribozyme, primer, and template, polymerization products were generated that implied the addition of G at five templating C positions. In the absence of added GTP, the polymerase ribozyme showed weak misincorporation of ATP on position +2, but no significant further extension (19). These results imply that multiple ribozyme-generated GTP molecules were incorporated by an RNA polymerase ribozyme into an RNA polymer, an important step toward modeling an RNA based metabolism.

## Discussion

This study describes the development of a GTP synthase ribozyme with a GTP turnover sevenfold higher than its parent. This improvement was achieved by a doped selection in emulsion that metabolically coupled ribozyme-catalyzed GTP synthesis to ribozyme-catalyzed primer extension, covered about 1.2 × 10^13^ variants of the parent GTP synthase ribozyme, and included the majority of up to five-mutation variants. HTS analysis and biochemical analysis identified the most efficient ribozyme (GTR1e) with 19 mutations, and a GTP turnover of 10-13. Kinetic analysis identified an almost threefold reduction in the apparent K_M_^APP^(cTmp) to 11 mM, while the K_M_^APP^ for guanosine remained unchanged at 1 mM. When coupled to an RNA polymerase ribozyme, up to five GTP molecules synthesized by GTR1e were incorporated into an RNA polymer, which represents an important step in testing how early RNA systems could have replicated from prebiotically available compounds.

How close is GTR1e to supporting a self-replicating RNA system? The GTR1e ribozyme itself contains 36 guanosines, and the currently most advanced RNA polymerase ribozyme contains 48 guanosines (16). Assuming that a total of five ribozymes with similar size are the minimum size of a self-replicating RNA system, about 400 GTP molecules would be required for each replication cycle, which is 30-fold higher than the GTP turnover of GTR1e. While two previous studies have shown multiturnover catalysis of small molecule substrates for ribozymes (31, 32), the current study and its precursor (19) generate such ribozymes with relevance for a self-replicating RNA system. Therefore, the reported sevenfold increase over the previous ribozyme represents a significant step toward a metabolism of self-replicating ribozymes.

Three of the four most active GTR1 variants originated from the selection line SH, which used small emulsion droplets and a highly active polymerase ribozyme variant. In other words, this line used the lowest selection pressure for high turnover but achieved the highest turnover. Why did the selection lines LH and LL not result in more active variants than SH? It is possible that the answer lies in the bottlenecks caused by high selection pressure (33): At higher selection pressure, the population size can decrease to a few copies per active sequence, which can lead to the stochastic removal of otherwise highly active sequences (34). In line with this explanation, the selection lines with large droplets (LL and LH) sampled a total of 188 pmol of the doped RNA library (1.6-fold coverage of the complexity), whereas the selection line with small droplets (SH) sampled a total of 3,750 pmol of the doped RNA library (31-fold coverage of the complexity). The selection lines with larger droplets (LL and LH) necessitated a smaller starting population to keep the total volume of the emulsion to a technically manageable size, and therefore increased the chance for the stochastic elimination of fit sequences. A second possibility is that the droplet diameter in lines LL and LH may have been larger than expected, or less homogeneous than expected (24), which could have allowed parasitic RNAs to have a stronger influence on the selection outcome (35).

Why does the rate of GTP synthesis slow down after about 4 h (Fig. 3 and *SI Appendix*, Figs S7, S11, and S12)? Since the RNA, cTmp, and guanosine by themselves have longer life times under these conditions, we assume that interactions between these molecules lead to a slowing of the reaction. An answer may come from the reaction kinetics at different concentrations of guanosine and cTmp: While different guanosine concentrations did not affect the ribozyme’s time of activity in the reaction (*SI Appendix*, Fig. S12), a decrease in cTmp concentration increased the life time from 4 to 5, 6, and 10 h when the cTmp concentration decreased from 100 mM to 50 mM, 25 mM, and 10 mM, respectively (*SI Appendix*, Fig. S11). This suggests that cTmp directly or indirectly inhibits ribozyme activity. We are currently studying the mechanism of this inhibition in detail and speculate that further evolution of the ribozyme will be able to increase its lifetime of GTP synthesis.

Can additional mutations in GTR1e lead to a further increase of GTP turnover? The beneficial mutations acquired in the current doped selection may still be far from the optimal sequence of GTR1 variants. Our calculation of mutation coverage suggested that the starting library covered only a small fraction of ribozyme variants with more than 6 mutations (*SI Appendix*, Fig. S4). While the testing of point mutations may have resulted in a minor benefit of a single, additional mutation (U64C, *SI Appendix*, Fig. S7*E*), further combinatorial studies may bring significant improvements from cooperative motifs of mutations. In comparison, the RNA polymerase ribozyme added a secondary structure motif over many cycles of mutagenesis and selection between 38 and 52 cycles of its ongoing in vitro evolution (36), which significantly improved its activity. The GTP synthase GTR1e has so far undergone “only” a total of 21 rounds of in vitro selection/evolution, with 18 rounds in the earlier study and 3 selection rounds (for line SH) in the present study. In addition to adding cycles of mutagenesis and selection, cooperative mutations may be found in a repeated doped selection as done in this study but with GTR1e as parent sequence, the addition of designed and/or randomized sequences in critical positions (14, 15) and changes in the design of the selection procedure (15, 16, 37). Future variants of GTR1e may display sufficient GTP turnover for a small self-replicating RNA system.

## Materials and Methods

### Generating the Doped Library.

The doped library was generated by PCR from a DNA ultramer 5′-ACATCACCGCCTTGTATGCACGGGATGGTCCTTGAAGTGTGCAGCCCTGACAACTCCTGGCCACTTCATAGACGCTGATGTTCTGAATCTCGCCCCTAACCATCTTCGGGATTCTCCAGAACCTCGCTCGCGCATGTAAGTCTCAACTCC-3′. The underlined region was doped with 2% of each mutation and 94% of the parent nucleotide as above. The parent sequence as shown above is an internally truncated version of GTR1 (*SI Appendix*, Fig. S2). The DNA ultramer was amplified for 9 cycles of PCR with the 5′-PCR primer AATT*TAATACGACTCACTATA*GGGATGTTGCTGACGAGCTAAGCGAAACTGCGGAAACGCAGTCCAACATCACCGCCTTGTAT to add the T7 promoter (italics), and the hammerhead ribozyme (underlined) at its 5′-terminus. The 3′-PCR primer gtggcggagagagggggatttgaaccccctctctccAGTTGGTTTGATGGAGTTGAGACTTACAT added the M1 RNase P recognition site at its 3′-terminus (lowercase). An estimated 90% of the template was PCR-amplifiable as judged by comparing the band intensity of ethidium bromide stained agarose gels from purified PCR products of known concentration. A total of 1.58 nmol of PCR amplifiable template was used for this PCR using *Taq* DNA polymerase. A total of 475 pmol of PCR products were used for transcription of the RNA prelibrary (i.e., before 3′-end processing) was generated by in vitro transcription using T7 RNA polymerase and purified by denaturing 7 M urea 5% PAGE.

#### 3′-end processing of the library.

The library RNAs were cleaved by the M1 RNase P ribozyme in vitro at their 3′-terminus to generate 2′, 3′-termini (*SI Appendix*, Fig. S3). This was done in a single-turnover reaction in trans as described previously (38). The M1 RNA was appended with the recognition site for the GTR1 library 3′-tail by PCR amplifying the M1 RNase P sequence with 5′ PCR primer AATTTAATACGACTCACTATAGGGTCAGCT and 3′ PCR primer AGGTGAAACTGACCGATAAGCCGGGTTCTG. The PCR product was transcribed and gel purified by denaturing 7 M urea 5% PAGE. The cleavage was set up by preincubating 1 µM of prelibrary with 50 mM Tris/HCl pH 7.9, 10 mM MgCl_2_, 800 mM NH_4_Cl, and 1.2 µM of M1 RNase P at 80 °C for 2 min, then at 50 °C for 10 min, then 22 °C for 5 min. This mixture was added to the same volume of reaction buffer containing 50 mM MES/KOH pH 5.1, 20 mM MgCl_2_, and 800 mM NH_4_Cl and incubated at 55 °C for 1 h. The processed product was then gel-purified by denaturing 7 M urea 5% PAGE. During this PAGE purification, the processed RNA library was recovered as well as the M1 RNA; the latter was reused for processing more prelibrary RNA.

#### In emulsio selection procedure.

The in emulsio selection procedure was conducted as described previously (19), with minor modifications (*SI Appendix*, Fig. S1). The processed library was preincubated with polymerase ribozyme in water at 80 °C for 2 min and then cooled on ice for 5 min. The annealed ribozymes were then mixed with the triphosphorylation buffer, to result in a final concentration of 50 mM Tris/HCl pH 7.9, 150 mM MgCl_2_, 200 mM KCl, 1 mM 6sG, 50 mM cTmp, 15 nM polymerase ribozyme and 5 nM doped GTR1 library RNA. Starting with the second selection round in selection lines LH and LL, the polymerase ribozyme and pool concentration were reduced to half. The SH selection line used a concentration of 0.5 µM doped GTR1 library RNA and 1.5 µM polymerase ribozyme in the aqueous phase. To generate the emulsion (24), the oil phase was prepared by mixing 4% (v/v) of the emulsifier ABIL EM90 (Evonik) with 96% (v/v) heavy mineral oil (O122-1, Fisher Scientific), and stirred for at least 15 min at room temperature. This oil phase was degassed in an oil vacuum and cooled in an ice bath while stirring for 10 min. The aqueous phase was added to the oil phase and stirred for 5 min to generate a raw emulsion. The raw emulsion was passed 7 times through a microfluidizer (Microfluidics, model M110L) at 4,000 psi with a 0.2 mm orifice shearing chamber (H30Z) for a 400 nm droplet diameter, and at 6,000 psi with a 0.1 mm orifice shear chamber (H10Z) for a 150 nm droplet diameter. The 150 nm emulsification was cooled on ice for 2 min after each pass to reduce heating of the emulsion, while this was not necessary for the 400 nm emulsion, where the temperature after 7 passes was at 35 °C. This emulsion was incubated at room temperature for 1 h.

To recover the RNA from the emulsion, more than one volume of ethyl ether was mixed with the emulsion. After centrifugating at 18,000 rpm for 1 h, the supernatant was removed, and the step was repeated. A white pellet had formed within the aqueous layer, which was dissolved in an equal volume of phenol and centrifugated. The combined aqueous phases were extracted with an equal volume of chloroform and centrifuged for 10 min. The top layer was collected and subjected to ethanol precipitation to recover the RNAs as large pellet. To remove the salt from the RNA pellet, the dissolved RNA pellet was subjected to ultrafiltration by size exclusion centrifugation columns (Pierce, 50 K MWCO, 0.5 mL, 88504) and collected by ethanol precipitation. The recovered RNA was loaded on aminophenyl mercury polyacrylaide gels (APM PAGE) to extract the tagged pools. The APM gel was made in four layers: The bottom layer (7 M urea, 5% PAGE) was poured horizontally to seal the plate sandwich, while the remaining layers were poured by standing the gel sandwich upright to obtain horizontal interfaces. All layers consisted of 7 M urea and 5% polyacrylamide except the second layer from the top, which also contained 80 µM aminophenyl mercury acrylamide. The extracted RNA library was separated on these gels, the thin interface of the top two layers was excised, and eluted with 300 mM NaCl and 5 mM DTT to obtain thio-tagged RNA library molecules. The extracted RNAs were reverse transcribed by Superscript IV (Thermo-Fisher) according to the manufacturer’s instructions, and PCR amplified as described for the generation of the RNA prelibrary.

#### HTS analysis.

The PCR products from each round of selection were appended with Illumina adapter and barcode sequences using PCR, and submitted to Illumina sequencing (NovaSeq X Plus 10B; UCSD IGM Genomics Center). The resulting sequences were filtered in R scripts for those with 100 to 120 nucleotides of the doped region and for the presence of the conserved region (39). The analysis was focused on the final rounds of the three selection lines: Round 9 in lines LL (2.3 million reads after filtering), LH (1.5 million reads after filtering), and round 3 in line SH (1.5 million reads after filtering). The top 10 abundant sequences of each line were identified. A Venn-diagram (*SI Appendix*, Fig. S6) identified 19 unique sequences that covered the top 10 sequences in all three lines [counted by Fastaptamer 2.0 (40)], one of them the parent (Par). All 15 sequences that appeared in the top two of at least one selection line, and/or in the top 10 in more than one line, were ordered as gBlocks (IDTDNA) and tested biochemically.

To analyze the enrichment of individual mutations or the covariation of multiple mutations, only sequences were considered that showed the designed length of 116 nucleotides for the doped region (*SI Appendix*, section 3). At least 1 million filtered reads were used for each selection round. Single mutations were analyzed based on each nucleotide’s enrichment over the frequency in the original library (E) (*SI Appendix*, Fig. S5). Covariation effects between the frequency of mutations A and B were calculated as covariation score CV = E(mutAB)/[E(mutA) × E(mutB)], where E(mutAB) is the enrichment of sequences that contained two mutations at the same time, and E(mutA) and E(mutB) represent the enrichment of sequences that contained each mutation. The epistasis values were then calculated as Epistasis = log_2_(CV_mut_/CV_wt_). The graphs in Fig. 2 and *SI Appendix*, Fig. S6 illustrate the epistasis scores by line thickness, where the line thickness is linearly related to the epistasis score. The line thickness of 5 corresponds to the highest identified epistasis score within a selection line, and a line thickness of 1 corresponds to the epistasis of the 20th most abundant epistasis in that selection line. Epistatic maps and epistatic values correlated with the secondary structure are shown in *SI Appendix*, Fig. S8.

#### In vitro analysis of GTP synthesis.

The selected and designed GTR variants were ordered from IDT as gBlocks, with a T7 promoter and a hammerhead ribozyme. The template was PCR amplified with 5′ primer AATTTAATACGACTCACTATAG and 3′ primer TGATGGAGTTGAGACTTACAT that generated the GTR1 variant without the polymerase binding site at the 3’ end. Polymerase ribozymes (25) were generated by PCR amplification from plasmids with 5′ primer AATTTAATACGACTCACTATAGGGTGGTGCCCTGACGAGCTAAGCGAAACTGCGGAAACGCAGTCGGCACCACAGTTGGTAC and 3′ primer GGAGCCGAAGCTCCGGG. The 5′-Cy5 labeled primer (IDTDNA) for the GTP assay had the sequence Cy5-CUCACCAACU-3′. For the GTP synthesis assay, the GTR variant RNA was renatured in the series of 10 min at 50 °C, 5 min at 22 °C, then added to the reaction buffer leading to final concentration of 50 mM Tris/HCl pH 7.9, 300 mM MgCl_2_, 1 M KCl, 200 mM Na_3_cTmp, 2 mM G, and 9 µM GTR. While G was added as dry powder/suspension up to Fig. 3, it was added as a 200 mM solution in DMSO for experiments in Figs. 4 and 5, which resulted in a 1% (v/v) concentration of DMSO in the GTP synthesis reaction. This DMSO concentration appeared uncritically low for RNA structure and function (41). The mixture was incubated at 22 °C for 20 h. A sample was taken and diluted with the same reaction buffer (but not containing GTR) and quenched by adding a 10-fold molar excess of competitor DNAs that were complementary to GTR1 and leaving the mixture at room temperature for 15 min. An aliquot of diluted and quenched reaction mixture was added to a premix with heat denatured 1 µM polymerase ribozyme and 0.5 µM Cy5 labeled primer, 50 mM Tris/HCl pH 7.9, 200 mM MgCl_2_, 200 mM KCl, and 90- to 360-fold diluted GTR reaction mix. This primer extension/GTP detection reaction was incubated at 25 °C for 1 h and quenched by ethanol precipitation with an excess of Na_2_EDTA over Mg^2+^. The precipitated mixture was dissolved in formamide loading buffer and 5 µM DNA TTTGTACCAACTGTGGTGCC to separate the polymerase ribozyme from the extended primer, heated for 2 min at 80 °C, and loaded to a 7 M urea 20% PAGE. The separated primer extension products were visualized by a Typhoon imager (Cytivia) and quantitated by the software Quantity One (Bio-Rad). The fraction of extended primer FE was calculated as FE = (Primer extended)/[(primer unextended) + (primer extended)].

#### RNA polymerization with ribozyme-generated GTP.

The reaction was set up similar as before (19) but with the polymerase ribozyme 71-89 (16) and a template that included two repeats of the polymerase ribozyme’s favorite template sequence (14), and with reaction conditions adjusted for the 71-89 polymerase ribozyme. In short, in a volume of 10 μL a concentration of 9 μM GTR, 33 mM Tris/HCl pH 7.0, 67 mM KCl, 200 mM KCl, 1.3 mM G, and 133 mM Na_3_cTmp was incubated for 20 h at 22 °C. Separately, a mixture of 3.2 pmol 5′-Cy5-labeled RNA primer, 4 pmol template, and 4 pmol polymerase ribozyme 71-89 (*SI Appendix*, Fig. S6) was heated to 80 °C for 2 min and cooled to 25 °C, then mixed with a premix and the 10 μL GTP synthesis reaction to result in a 40 μL volume containing 80 nM Cy5-primer, 100 nM template, 100 nM 71-89, 0.2 mM UTP, CTP, ATP, 50 mM Tris/HCl pH 8.3, 50 mM MgCl_2_, 50 mM KCl, and 8% (w/v) PEG-8000. After incubation at 25 °C for the indicated times, 10 μL samples were quenched with 1.5 μL 500 mM Na_2_EDTA, ethanol precipitated, heat renatured in the presence of 8 pmol competitor DNA to resolve the primer/template duplex (19), and separated on a 7 M urea 20% PAGE. Quantitation of signals was done as described above.

## Supplementary Material

Appendix 01 (PDF)

## Data Availability

The high throughput sequencing data are available at NCBI’s SRA at submission ID (SUB16210974) under the BioProject ID: PRJNA1470371 (42). The code in R for filtering high throughput sequencing data is available in GitHub at the link https://github.com/xuhan9798/HTS-analysis-for-GTR1e-libraries (39). Other data are included in the article and/or *SI Appendix*.

## References

[1] A. Rich, “On the problems of evolution and biochemical information transfer” in Horizons in Biochemistry, M. Kasha, B. Pullman, Eds. (Academic Press, New York, NY, 1962). pp. 103–126.

[2] L. E. Orgel, Evolution of the genetic apparatus. J. Mol. Biol. **38**, 381–393 (1968). 5718557 10.1016/0022-2836(68)90393-8

[3] F. H. C. Crick, The origin of the genetic code. J. Mol. Biol. **38**, 367–379 (1968). 4887876 10.1016/0022-2836(68)90392-6

[4] C. R. Woese, The Genetic Code: The Molecular Basis for Genetic Expression (Harper & Row, New York, 1967).

[5] P. Nissen, J. Hansen, N. Ban, P. B. Moore, T. A. Steitz, The structural basis of ribosome activity in peptide bond synthesis. Science **289**, 920–930 (2000). 10937990 10.1126/science.289.5481.920

[6] H. B. White III, Coenzymes as fossils of an earlier metabolic state. J. Mol. Evol. **7**, 101–104 (1976). 1263263 10.1007/BF01732468

[7] P.-J. Kim , Metabolite essentiality elucidates robustness of *Escherichia coli* metabolism. Proc. Natl. Acad. Sci. U.S.A. **104**, 13638–13642 (2007). 17698812 10.1073/pnas.0703262104 PMC1947999

[8] S. A. Becker, N. D. Price, B. Ø. Palsson, Metabolite coupling in genome-scale metabolic networks. BMC Bioinformatics **7**, 111 (2006). 16519800 10.1186/1471-2105-7-111 PMC1420336

[9] D. Wacey, M. R. Kilburn, M. Saunders, J. Cliff, M. D. Brasier, Microfossils of sulphur-metabolizing cells in 3.4-billion-year-old rocks of Western Australia. Nat. Geosci. **4**, 698–702 (2011).

[10] N. Noffke, D. Christian, D. Wacey, R. M. Hazen, Microbially induced sedimentary structures recording an ancient ecosystem in the ca. 3.48 billion-year-old Dresser Formation, Pilbara, Western Australia. Astrobiology **13**, 1103–1124 (2013). 24205812 10.1089/ast.2013.1030 PMC3870916

[11] A. D. Ellington, J. W. Szostak, In vitro selection of RNA molecules that bind specific ligands. Nature **346**, 818–822 (1990). 1697402 10.1038/346818a0

[12] C. Tuerk, L. Gold, Systematic evolution of ligands by exponential enrichment: RNA ligands to bacteriophage T4 DNA polymerase. Science **249**, 505–510 (1990). 2200121 10.1126/science.2200121

[13] D. P. Bartel, J. W. Szostak, Isolation of new ribozymes from a large pool of random sequences. Science **261**, 1411–1418 (1993). 7690155 10.1126/science.7690155

[14] W. K. Johnston, P. J. Unrau, M. S. Lawrence, M. E. Glasner, D. P. Bartel, RNA-catalyzed RNA polymerization: Accurate and general RNA-templated primer extension. Science **292**, 1319–1325 (2001). 11358999 10.1126/science.1060786

[15] R. Cojocaru, P. J. Unrau, Processive RNA polymerization and promoter recognition in an RNA World. Science **371**, 1225–1232 (2021). 33737482 10.1126/science.abd9191

[16] N. Papastavrou, D. P. Horning, G. F. Joyce, RNA-catalyzed evolution of catalytic RNA. Proc. Natl. Acad. Sci. U.S.A. **121**, e2321592121 (2024). 38437533 10.1073/pnas.2321592121 PMC10945747

[17] E. Gianni , A polymerase ribozyme that can synthesize both itself and its complementary strand. bioXriv [Preprint] (2024), http://biorxiv.org/lookup/doi/10.1101/2024.10.11.617851 [Accessed 4 May 2025].

[18] K. Lohmann, Über die Pyrophosphatfraktion im Muskel. Naturwissenschaften **17**, 624–625 (1929).

[19] A. Akoopie, J. T. Arriola, D. Magde, U. F. Müller, A GTP-synthesizing ribozyme selected by metabolic coupling to an RNA polymerase ribozyme. Sci. Adv. **7**, eabj7487 (2021). 34613767 10.1126/sciadv.abj7487 PMC8494290

[20] Y. Yamagata, H. Watanabe, M. Saitoh, T. Namba, Volcanic production of polyphosphates and its relevance to prebiotic evolution. Nature **352**, 516–519 (1991). 11536483 10.1038/352516a0

[21] M. A. Pasek, T. P. Kee, D. E. Bryant, A. A. Pavlov, J. I. Lunine, Production of potentially prebiotic condensed phosphates by phosphorus redox chemistry. Angew. Chem. Int. Ed. Engl. **47**, 7918–7920 (2008). 18781567 10.1002/anie.200802145

[22] M. A. Pasek, J. P. Harnmeijer, R. Buick, M. Gull, Z. Atlas, Evidence for reactive reduced phosphorus species in the early Archean ocean. Proc. Natl. Acad. Sci. U.S.A. **110**, 10089–10094 (2013). 23733935 10.1073/pnas.1303904110 PMC3690879

[23] M. Pasek, K. Block, Lightning-induced reduction of phosphorus oxidation state. Nat. Geosci. **2**, 553–556 (2009).

[24] D. Magde, A. Akoopie, M. D. Magde, U. F. Müller, Water/oil emulsions with controlled droplet sizes for in vitro selection experiments. ACS Omega **6**, 21773–21783 (2021). 34471779 10.1021/acsomega.1c03445 PMC8388082

[25] A. Akoopie, U. F. Muller, The NTP binding site of the polymerase ribozyme. Nucleic Acids Res. **46**, 10589–10597 (2018). 30289487 10.1093/nar/gky898 PMC6237761

[26] G. L. Igloi, Interaction of tRNAs and of phosphorothioate-substituted nucleic acids with an organomercurial. Probing the chemical environment of thiolated residues by affinity electrophoresis. Biochemistry **27**, 3842–3849 (1988). 3044450 10.1021/bi00410a048

[27] P. J. Unrau, D. P. Bartel, RNA-catalysed nucleotide synthesis. Nature **395**, 260–263 (1998). 9751052 10.1038/26193

[28] E. Biondi, D. H. Burke, Separating and analyzing sulfur-containing RNAs with organomercury gels. Methods Mol. Biol. **883**, 111–120 (2012). 22589128 10.1007/978-1-61779-839-9_8

[29] E. Etaix, L. E. Orgel, Phosphorylation of nucleosides in aqueous solution using trimetaphosphate: Formation of nucleoside triphosphates. J. Carbohydr. Nucleosides Nucleotides **5**, 91–110 (1978).

[30] F. Chizzolini, A. D. Kent, L. F. M. Passalacqua, A. Lupták, Enzymatic RNA production from NTPs synthesized from nucleosides and trimetaphosphate. ChemBioChem **22**, 2098–2101 (2021). 33798271 10.1002/cbic.202100085

[31] B. Seelig, S. Keiper, F. Stuhlmann, A. Jäschke, Enantioselective ribozyme catalysis of a bimolecular cycloaddition reaction. Angew. Chem. Int. Ed. **39**, 4576–4579 (2000). 11169675

[32] M. M. Conn, J. R. Prudent, P. G. Schultz, Porphyrin metalation catalyzed by a small RNA molecule. J. Am. Chem. Soc. **118**, 7012–7013 (1996).

[33] Z. N. Amini, U. F. Muller, Low selection pressure aids the evolution of cooperative ribozyme mutations in cells. J. Biol. Chem. **288**, 33096–33106 (2013). 24089519 10.1074/jbc.M113.511469 PMC3829158

[34] P. G. Higgs, U. F. Muller, Principles of in vitro selection of ribozymes from random sequence libraries. J. R. Soc. Interface **22**, 20240878 (2025). 40233799 10.1098/rsif.2024.0878 PMC11999736

[35] Y. Bansho , Importance of parasite RNA species repression for prolonged translation-coupled RNA self-replication. Chem. Biol. **19**, 478–487 (2012). 22520754 10.1016/j.chembiol.2012.01.019

[36] X. Portillo, Y.-T. Huang, R. R. Breaker, D. P. Horning, G. F. Joyce, Witnessing the structural evolution of an RNA enzyme. Elife **10**, e71557 (2021). 34498588 10.7554/eLife.71557 PMC8460264

[37] J. Attwater, A. Raguram, A. S. Morgunov, E. Gianni, P. Holliger, Ribozyme-catalysed RNA synthesis using triplet building blocks. Elife **7**, e35255 (2018). 29759114 10.7554/eLife.35255 PMC6003772

[38] E. Kikovska, S. G. Svard, L. A. Kirsebom, Eukaryotic RNase P RNA mediates cleavage in the absence of protein. Proc. Natl. Acad. Sci. U.S.A. **104**, 2062–2067 (2007). 17284611 10.1073/pnas.0607326104 PMC1892975

[39] X. Han , HTS-analysis-for-GTR1e-libraries. GitHub. https://github.com/xuhan9798/HTS-analysis-for-GTR1e-libraries. Deposited 26 May 2026.

[40] S. T. Kramer, P. R. Gruenke, K. K. Alam, D. Xu, D. H. Burke, FASTAptameR 2.0: A web tool for combinatorial sequence selections. Mol. Ther. Nucleic. Acids **29**, 862–870 (2022). 36159593 10.1016/j.omtn.2022.08.030 PMC9464650

[41] J. Lee, C. E. Vogt, M. McBrairty, H. M. Al-Hashimi, Influence of dimethylsulfoxide on RNA structure and ligand binding. Anal. Chem. **85**, 9692–9698 (2013). 23987474 10.1021/ac402038t PMC3855037

[42] X. Han , GTR1e libraries. BioProject. http://www.ncbi.nlm.nih.gov/bioproject/1470371. Deposited 26 May 2026.

