# Supplementary material for: A GTP synthase ribozyme with increased GTP turnover
Source: Proc Natl Acad Sci U S A. 2026 Jun 9;123(24):e2520997123. doi: 10.1073/pnas.2520997123 (PMC13273283; doi:10.1073/pnas.2520997123)
Supplement: Supplementary file 1 — Appendix 01 (PDF) [file pnas.2520997123.sapp.pdf]

A GTP Synthase Ribozyme with increased GTP Turnover

Xu Han[1] , Zoe J. Pepper[1], Joshua T. Arriola[1],
and Ulrich F. Müller[1]*
[1]Department of Chemistry & Biochemistry, University of California San Diego, La Jolla, CA 92093
*corresponding author: ufmuller@ucsd.edu

Supplementary Information

S.I. 1: Novel aspects of the selection procedure 2

S.I. 2: Quantitative description of the selection in emulsion 3

S.I. 3: Analysis of mutation covariations and epistatic interactions 6

Figure S1: Emulsion droplet size and incubation time 7

Figure S2: Internal truncations of the parent ribozyme 8

Figure S3: Processing of the library by RNase P M1 RNA 9

Figure S4: Expected coverage of Mutations by the doped library 10

Figure S5: Frequency of doped mutations in the library 11

Figure S6: Selection of mutations and covariations 12

Figure S7: Biochemical identification of GTR1e 13

Figure S8: Epistatic interactions 14

Figure S9: Clustering analysis for an invalid epistatic network 15

Figure S10: Clustering analysis for two valid epistatic networks 16

Figure S11: Dependence on the cTmp concentration 17

Figure S12: Dependence on the guanosine concentration 18

References 19

S.I. 1: Novel aspects of the selection procedure

The selection procedure was based on the previously published procedure, with several modifications [1]. Three parallel selection lines were conducted, exploring specific adjustments to help select library sequences mediating higher GTP turnover.

First, the three lines of selection differed in their emulsion droplet size between 150 nm diameter as in the original selection (selection line SH) and an increased emulsion droplet diameter of about 400 nm diameter. This corresponds to a 20-fold difference in droplet volume, which increased the selection pressure for multiple turnover (Fig. S1). Second, the concentrations of the substrates 6sG and cTmp were kept high throughout the selection (1 mM and 50 mM, respectively). This avoided the need for strong substrate binding and thereby reduced the danger of product inhibition. Third, a 7-fold less active variant of the polymerase ribozyme was used to tag the successful catalysts with 6sGTP. This was accomplished by using a polymerase ribozyme mutant with a C in the NTP binding loop at position 156 as opposed to a U [2]. This mutation required more 6sGTP for tagging, and thereby increased the selection pressure on multiple turnover. The selection line LL employed a large droplet diameter and low activity polymerase ribozyme, line LH employed a large droplet diameter and a highly active polymerase ribozyme, and line SH employed a small droplet diameter and a highly active polymerase ribozyme.

Four additional modifications were made to help the selection procedure. First, the ribozyme was truncated at an internal stem-loop while maintaining activity (Fig. 1 and Fig. S2). This reduced the size of the partially randomized region of the ribozyme, and thereby allowed better sampling of the ribozyme's sequence variants. Second, the 3'-terminus of the ribozyme library was processed by the M1 RNase P ribozyme instead of the previously used DNAzyme to generate a more homogeneous 3'-terminus due to more site-specific cleavage by the M1 RNA (Fig. S3). Third, an additional linker was inserted between the ribozyme library and the annealing site to the polymerase ribozyme to reduce the chance of the GTP synthase becoming dependent on the structure of the polymerase ribozyme (Fig. 1). Fourth, the emulsion was incubated for only 1 hour because this was expected to lead to the best discrimination between background reaction, the activity of the parent ribozyme, and a desired GTP synthase with increased turnover (Fig. S1). The first round of the selection covered an effective complexity of 1.2×10^{13} sequences (see materials and methods). With a doping ratio of 6% in the 116 partially randomized positions of the library (94% parent sequence and 2% of each mutation), we expected this complexity to cover all single-, double-, triple, quadruple- and pentuple mutations in this region (Fig. S4). The two selection lines with larger emulsion droplets (LH and LL) differed in the activity of the polymerase ribozyme due to a mutation in the NTP binding loop of the polymerase ribozyme, with a C for low activity (LL) and a U for high activity (LH) (Fig. 1, [2]). Therefore, the selection pressure for high 6sGTP turnover was highest in line LL with large emulsion droplets and low polymerase activity, intermediate in line LH with large emulsion droplets and high polymerase activity, and lowest in line SH with small emulsion droplets and high polymerase activity. Three rounds of selection were conducted with selection line SH, and nine rounds of selection were conducted with the selection lines LH and LL.

S.I. 2: Quantitative aspects of the selection in emulsion

The selection conditions were tuned for the goal of selecting GTP synthase ribozymes with increased GTP turnover. The parameters included (1) the emulsion droplet volume, (2) the activity of the polymerase ribozyme that was used for tagging, (3) the concentration of the substrates 6-thio guanosine and cyclic trimetaphosphate, and (4) the incubation time of the coupled reaction in the droplets. In addition, (5) stochastic effects were considered. The five sections below discuss each of these five points.

(1) Droplet volume: (Fig. S1E-F)

The selection experiments were set up such that the majority of library molecules were alone in their emulsion droplet to reduce the selection of inactive molecules. The concentration of library molecules in the aqueous phase needed to be low enough to reduce multiple library molecules per droplet, while keeping it high enough to cover a large combinatorial complexity with a reasonable emulsion volume. Because the droplet sizes differed between the three lines of selection (SH, LH, LL), the library concentration used for the small emulsion droplets (line SH; droplet diameter 150 nm) was higher at 0.5 µM, while a concentration of 5 nM library was used for the larger emulsion droplets (lines LH and LL; droplet diameter 400 nm). The control of these droplet diameters was achieved by using defined shearing forces during the emulsification procedure, and by repeating the shearing step seven times to achieve a narrow droplet size distribution [3]. The droplet volume $V=4/3\ r^3\pi$ for the droplet diameter of d=150 nm (r=d/2) is $1.77\times10^{-21}\ m^3$, therefore the concentration for a single molecule per droplet in this emulsion $1/(V\times N_A)$ = 940 nM. Because the selection line SH with 150 nm diameter droplets used a library concentration of 500 nM, it contained an average ratio of 0.532 library molecules per droplet. In contrast, the droplet volume V for the droplet diameter of d=400 nm is $V=4/3\ r^3\pi = 3.35\times10^{-20}\ m^3$, and the concentration for a single molecule per droplet in this emulsion $1/(V\times N_A)$ = 50 nM. Because the selection lines with 400 nm diameter droplets (LH, LL) used a library concentration of 5.0 nM, they contained an average of 0.101 library molecules per droplet.

The statistical distribution of library molecules per droplet is given by the Poisson distribution, with $P(K) = (L^K e^{-L})/K!$ where L is the average ratio of library molecules per droplet (here L=0.532 or 0.101) and K is the specific number of molecules per droplet.

For the 150 nm droplets, 59% of the library molecules were alone on their droplet (K=1), 31% together with a second library molecule (K=2), 8.3% together with two more library molecules (K=3), and 1.5% together with three more library molecules (K=4). Less than 0.2% of the library molecules shared their droplet with more than three other library molecules (K>4). In later selection rounds, the library molecule concentration was reduced 2-fold, resulting in 77% of library molecules alone in their droplet, 20% of library molecules sharing their droplet with one more library molecule, and 3.0% sharing their droplet with two other library molecules.

For the droplet diameter of 400 nm and the average ratio of 0.101 library molecules per droplet, 90% of the library molecules were alone in the droplet, 9% together with a second library molecule, and 0.5% with more than one additional library molecule. In the second, and later selection rounds, where the library concentration was reduced 2-fold, 95% of the library molecules were alone in their droplet and 5% with one or more additional library molecules.

This setup reduces the emergence of molecular parasites: Since selection rounds 2 and later have 77% (150 nm droplets) and 95% (400 nm droplets) of their library molecules alone in their droplet, this generates at least a 4-fold and 20-fold disadvantage for library molecules that depend on other library molecules for 6sGTP synthesis.

Quantitative aspects of the selection in emulsion - continued

Bulk experiments with 6sGTP at 10 nM, 100 nM, and 1,000 nM suggested that the highly active polymerase ribozyme in a 150 nm droplet would tag slightly less than 43% of the library molecules after 1 hour of incubation (Fig. S1), whereas in a 400 nm diameter droplet, it would tag about 0.05% of the library molecules – if only a single single 6sGTP molecule was generated within the droplet. For multiple-turnover library RNAs (i.e. ribozymes catalyzing multiple turnover 6sGTP synthesis), the average fraction of tagging should increase with the number N of 6sGTP molecules generated, which can be calculated with the equation $TE=(100\%-(99.95\%)^N$, where TE is the tagging efficiency. For example, a turnover N of 10, or 100 should increase the tagging efficiency TE in 400 nm diameter droplets from 0.05% to about 0.5%, and 4.9%, respectively. In 150 nm diameter droplets, the tagging efficiency should reach 64% with a turnover of 2, 78% with a turnover of 3, 92% with a turnover of 5, and more than 99% with a turnover of 10.

(2) Polymerase ribozyme / tagging ribozyme activity

The tagging efficiency in 400 nm diameter droplets with a 'low activity polymerase ribozyme' is about 7-fold lower, which further increased the selection pressure for multiple turnover: The 'high activity polymerase/tagging ribozyme' (selection lines SH and LH) carried the mutations A156U, C79U, and C113U, which were optimized for using 6sGTP as substrate [2]. In contrast, selection line LL used the 'low activity polymerase / tagging ribozyme' with mutations A156C, C79U, and C113U, which mediate a 7-fold lower activity with 6sGTP. Given that the 6sGTP concentration was already limiting for the 400 nm diameter droplets, the tagging efficiency in large droplets with the lower activity polymerase ribozyme increased the selection stringency to an extreme, so that a turnover of about 100 would be required to select 0.7%, and a turnover of 1,000 would be required for the selection of 5.6% of the molecules with that activity. Therefore, the three lines of selection were tuned for optimizing specific ranges of 6sGTP turnover: Line SH (150 nm diameter droplets, high activity polymerase) was well-suited to select GTP synthase ribozymes with a turnover of about 5, whereas selection lines LH and LL were suited to select GTP synthase ribozymes for GTP turnovers between 100 and 1,000.

(3) Concentration of the substrates 6-thio guanosine (6sG) and cyclic trimetaphosphate (cTmp)

We hypothesized that the GTP synthase ribozyme GTR1 that was selected in the original selection [1] was in part limited to low turnover because during the selection, the substrate concentrations were successively reduced. The the first two selection rounds, the concentration of cTmp and 6sG were 50 mM and 1 mM, respectively, while the last selection round 18 contained 100-fold less (0.5mM) cTmp and 20-fold less (0.05 mM) 6sG. The low substrate concentrations may have enriched the population for ribozyme variants that bound both substrates tightly, with the consequence that the reaction product 6sGTP may also have been bound tightly. This would reduce product release and thereby the turnover of the reaction. For this reason, the concentration of both substrates cTmp and 6sG was kept high at 50 mM and 1 mM, respectively. We believe that the success of selecting higher-turnover GTP synthases in the current study was in part due to these high substrate concentrations.

Quantitative aspects of the selection in emulsion - continued

<u>(4) Incubation time for the metabolically coupled reaction</u>
The incubation time of the coupled reaction in emulsion strongly affects the enrichment of multi-turnover ribozymes. As shown in pilot experiments (Fig. S1 A-D), the uncatalyzed reaction between 50 mM cTmp and 1 mM 6sG leads to tagging of about 2.7% of the library molecules per hour. At a concentration of 1 μM 6sGTP, 62% of the ribozyme were tagged after 1 hour, more than 20 fold higher than the background reaction. In a coupled GTP synthesis reaction with the parent GTR1 ribozyme, about 7.5% of the RNA were tagged within one hour. These results show that reaction times longer than one hour were not promising for enriching multiple turnover ribozymes. Short reaction times have the disadvantage that the coupled reaction relies on two reaction kinetics: 6sG triphosphorylation, and 6sGTP tagging. Before the selection, no high-turnover GTP synthase was available to test how much shorter than 1 hour would be a promising reaction time. Additionally, the emulsification process took about 15 minutes, and reaction times shorter than one hour might show effects that were dominated by reactions in the partially processed emulsion rather than the emulsion with narrow droplet size distribution. Therefore, a reaction time was chosen as promising time for the incubation of the metabolically coupled reactions in emulsion.

<u>(5) Statistical effects:</u> In order to increase the chances of finding highly active sequences, each sequence of the initial PCR library was covered less than 10-fold. This low representation made it possible to cover a larger number of different sequences, some of which may have been in the winning cluster of the selection (see Fig.S5). However, this low representation comes with a trade-off, which is an increase in statistical effects for individual sequences. A mathematical description, and computational simulations of this effect were published recently [4].

S.I. 3: Analysis of mutation covariations and epistatic interactions

To analyze the enrichment of individual mutations or the covariation of multiple mutations, only sequences were considered with the designed length of 116 nucleotides for the doped region. At least 1 million filtered reads were analyzed for each selection round. Each selection line (SH,LH,LL) was analyzed separately.

The **fraction of reads (f)** was used to calculate the abundance of a sequence in each selection round with

$$f = N_{seq} / N_{total}$$

where N_{seq} is the number of reads of a sequence in a selection round and N_{total} is the number of total reads analyzed in that selection round.

The **Enrichment (E)** of a given sequence or mutation was calculated as the ratio between the frequency in the last selection round compared to the frequency in the doped library as

$$E = f_{Rlast} / f_{R0}$$

where f_{Rlast} is the fraction of a sequence in the last round of selection and f_{R0} is the fraction of that sequence in the original library (round 0). Taking this ratio as opposed to the frequency in the last selection round was necessary because the doped library had a significant nucleotide bias in the doping ratio (Fig. S5).

The **Fitness** of single mutation over the wildtype sequence was calculated as in McRae et al. [5] with

$$\text{Fitness} = \log_2(E_{mut} / E_{wt})$$

where E_{mut} is the enrichment of sequences that contain a specific mutation, and E_{wt} is the enrichment of sequences that contain the wildtype nucleotide at that position.

Covariation effects (CV) between two signal mutations (eg. A and B) were analyzed based on the enrichment of sequences containing both mutations (AB) using the equation

$$CV = E_{mutAB} / [E_{mutA} \times E_{mutB}]$$

where E_{mutAB} is the enrichment of sequences that contain both mutations, while E_{mutA} and E_{mutB} represent the enrichment of sequences that contain A mutation or B mutation respectively.

The **Epistasis** was used to calculate the significance of mutation covariations compared with the wildtype sequence as in [5],using the equation

$$\text{Epistasis} = \text{Fitness}_{AB} - \text{Fitness}_{A} - \text{Fitness}_{B} = \log_2(CV_{mut} / CV_{wt})$$

where CV_{mut} is the covariation effects of two possible mutations and CV_{wt} is the covariation effects of the original nucleotides at those two positions.

The epistasis and fitness calculations are as reported [5], with the only difference that the selection in the current study was not done in triplicate, therefore no errors could be assigned to calculated values.

Figure S1

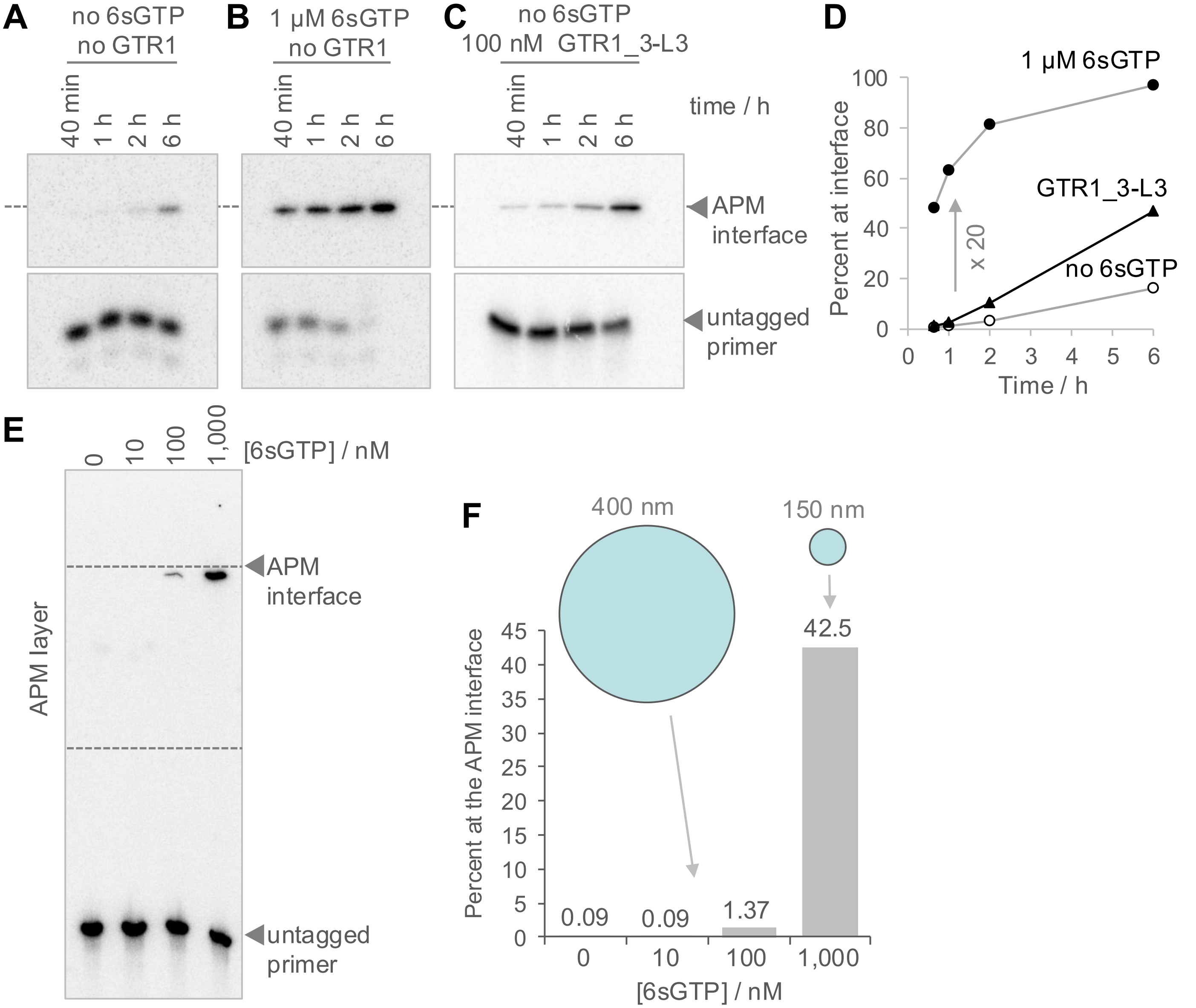

Figure S1: Emulsion droplet size and incubation time. Shown is the tagging of a primer with 6sGTP with a polymerase ribozyme under different conditions (for a schematic see figure 3A), and the retention of the tagged primer at the interface of a denaturing APM PAGE. **(A-C)** Dependence of 6sGTP tagging efficiency on the tagging time. The images show autoradiograms of 5'-[^{32}P] radiolabeled primers that were extended with 6-thio GTP (6sGTP) and separated by 7M urea 20% APM PAGE. The reaction shown in (A) contained only the substrates of 1 mM 6sG and 50 mM Na_3cTmp under reaction conditions ('no 6sGTP'), (B) the reaction mixture but with the addition of 1 µM 6sGTP, or (C) the same reaction mixture but with 6sG, cTmp and the parent GTP synthetase ribozyme GTR1_3-L3 ('L3' indicates the linker sequence between GTR1 and the 3'-terminus that was used in the selection). The gels contained 80 µM aminophenyl mercury (APM) in the APM layer, with the APM interface indicated with a horizontal dashed line. The reaction time of the 6sGTP synthesis reaction is indicated. The primer has the same sequence as the RNA library 3'-terminus during the selection, therefore the shown reaction mimicks of library RNAs tagging under selection conditions. **(D)** Graph showing the quantified signals from (A-C). The 1 hour reaction time shows the largest signal enhancement between the GTR1_3 catalyzed reaction and the reaction with 1 µM 6sGTP, a 20-fold increase in signal. This 20x increase shows that there is a significant selective advantage for GTR1 variants with higher GTP turnover. **(E)** Dependence of the tagging reaction on 6sGTP concentration. Shown is an autoradiogram of a 7M urea 5% PAGE separation with 80 µM APM in the APM layer. All reactions were incubated for 1 hour. **(F)** Graph showing the results in (E) and illustrating the effects of droplet size on 6sGTP concentration. The 1 µM 6sGTP corresponds to one molecule per 150 nm droplet, the 50 nM 6sGTP corresponds to one molecule per 400 nm droplet.

Figure S2

A

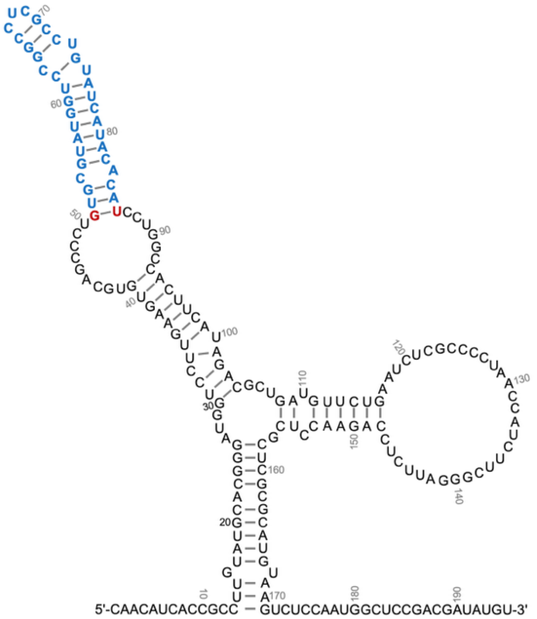

C

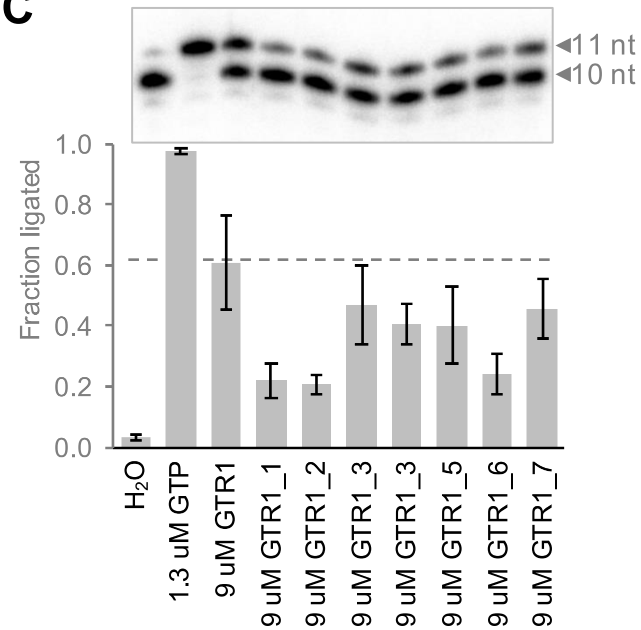

B

GTR1 CAACAUCACCGCCUUGUAUGCACGGGAUGGUCCUUGAAGUGUGCAGCCCUGUGCGUAUGGUCCGGCCUCGCCUGUAUCAUACACAUCCUG
GCCACUUCAUAGACGCUGAUGUUCUGAAUCUCGCCCCUAACCAUCUUCGGGAUUCUCCAGAACCUCGCUCGCGCAUGUAAGUCUC-3'

GTR1_1 CAACAUCACCGCCUUGUAUGCACGGGAUGGUCCUUGAAGUGUGCAGCCCUG__________CACAAC____________________UCCUG
GCCACUUCAUAGACGCUGAUGUUCUGAAUCUCGCCCCUAACCAUCUUCGGGAUUCUCCAGAACCUCGCUCGCGCAUGUAAGUCUC-3'

GTR1_2 CAACAUCACCGCCUUGUAUGCACGGGAUGGUCCUUGAAGUGUGCAGCCCUG__________AACAACAACA______________UCCUG
GCCACUUCAUAGACGCUGAUGUUCUGAAUCUCGCCCCUAACCAUCUUCGGGAUUCUCCAGAACCUCGCUCGCGCAUGUAAGUCUC-3'

GTR1_3 CAACAUCACCGCCUUGUAUGCACGGGAUGGUCCUUGAAGUGUGCAGCCCUG__________ACAAC_____________________UCCUG
GCCACUUCAUAGACGCUGAUGUUCUGAAUCUCGCCCCUAACCAUCUUCGGGAUUCUCCAGAACCUCGCUCGCGCAUGUAAGUCUC-3'

GTR1_4 CAACAUCACCGCCUUGUAUGCACGGGAUGGUCCUUGAAGUGUGCAGCCCUG__________ACAC______________________UCCUG
GCCACUUCAUAGACGCUGAUGUUCUGAAUCUCGCCCCUAACCAUCUUCGGGAUUCUCCAGAACCUCGCUCGCGCAUGUAAGUCUC-3'

GTR1_5 CAACAUCACCGCCUUGUAUGCACGGGAUGGUCCUUGAAGUGUGCAGCCCUG__________ACA_______________________UCCUG
GCCACUUCAUAGACGCUGAUGUUCUGAAUCUCGCCCCUAACCAUCUUCGGGAUUCUCCAGAACCUCGCUCGCGCAUGUAAGUCUC-3'

GTR1_6 CAACAUCACCGCCUUGUAUGCACGGGAUGGUCCUUGAAGUGUGCAGCCCUG__________CA________________________UCCUG
GCCACUUCAUAGACGCUGAUGUUCUGAAUCUCGCCCCUAACCAUCUUCGGGAUUCUCCAGAACCUCGCUCGCGCAUGUAAGUCUC-3'

GTR1_7 CAACAUCACCGCCUUGUAUGCACGGGAUGGUCCUUGAAGUGUGCAGCCCUGUG_________UGGAA______________ACACAUCCUG
GCCACUUCAUAGACGCUGAUGUUCUGAAUCUCGCCCCUAACCAUCUUCGGGAUUCUCCAGAACCUCGCUCGCGCAUGUAAGUCUC-3'

Figure S2. Internal truncations of the GTR1 ribozyme to identify a shorter GTR1 variant as parent ribozyme for the doped selection. **(A)** Secondary structure based on Akoopie et al. [1]. Based on this secondary structure and sequence conservation in the previous selection, the stem-loop between G51 (red) and U86 (red) was replaced with A & C-rich inserts (blue) that would be able to span the gap that was left by removing the duplex, and were predicted by mfold to not form a strong secondary structure. **(B)** Sequences of the parent ribozyme and the seven variants where the stem-loop was replaced with a shorter insert. **(C)** Activity assay of GTR1 and its seven truncation variants (see figure 3A). Shown on top is the scan of a denaturing polyacrylamide gel after separating the products of the assay. Extensions of the 10 nucleotide primer correspond to the detection on GTP, which was either added directly (1.3 uM GTP) or produced by the GTR and consecutive dilution into the assay. Because GTR1_3 showed the highest average activity of all truncation variants and it was within error of GTR1, it was chosen as the parent construct. Error bars are standard deviations from triplicate experiments.

Figure S3

A

Library RNA before processing: 5'-
CAACAUCACCGCCUUGUAUGCACGGGAUGGUCCUUGAAGUGUGCAGCCCUG**ACAAC**UCCUGGCC
ACUUCAUAGACGCUGAUGUUCUGAAUCUCGCCCCUAACCAUCUUCGGGAUUCUCCAGAACCUCGC
UCGCGCAUGUAAGUCUCAACUCCAUCAAACCAACUGGAGAGAGGGGUUCAAAUCCCCCUCUCUC
CGCCAC-3'

RNase P M1 RNA: 5'-
GAAGCUGACCAGACAGUCGCCGCUUCGUCGUCGUCCUCUUCGGGGGA
GACGGGCGGAGGGGAGGAAAGUCCGGGCUCCAUAGGGCAGGGUGCCA
GGUAACGCCUGGGGGGGAAACCCACGACCAGUGCAACAGAGAGCAAAC
CGCCGAUGGCCCGCGCAAGCGGGAUCAGGUAAGGGUGAAAGGGUGCG
GUAAGAGCGCACCGCGCGGCUGGUAACAGUCCGUGGCACGGUAAACUC
CACCCGGAGCAAGGCCAAAUAGGGGUUCAUAAGGUACGGCCCGUACUG
AACCCGGGUAGGCUGCUUGAGCCAGUGAGCGAUUGCUGGCCUAGAUGA
AUGACUGUCCACGACAGAACCCGGCUUAUCGGUCAGUUUCACCU-3'

Library RNA after processing:
5'-
CAACAUCACCGCCUUGUAUGCACGGGAUGGUCCUUGAAGUGUGCAGCCCUG**ACAAC**UCCUGGCC
ACUUCAUAGACGCUGAUGUUCUGAAUCUCGCCCCUAACCAUCUUCGGGAUUCUCCAGAACCUCGC
UCGCGCAUGUAAGUCUCAACUCCAUCAAACCAACU-3'

B

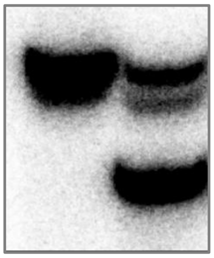

5% denaturing PAGE

C

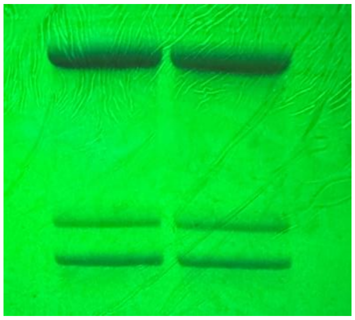

5% denaturing PAGE

Figure S3. Processing of the library 3'-termini by RNase P M1 RNA. **(A)** Sequences of the RNAs involved in the processing reaction. The Library RNA sequence before processing and after processing, and the sequence of the RNase P M1 RNA are annotated. The doped region of the library construct is shown in teal, with the truncated insert in bold.The linker sequence is in red, the primer sequence that binds to the polymerase ribozyme in blue, and the recognition sequence for the RNase P1 M1 RNA in green. This recognition sequence forms a strong 13-base pair duplex that is capped by a GU pair at the cleavage site. The recognition sequence mimicks the tRNA acceptor stem, with a 3'-terminal CCA sequence. **(B)** Autoradiogram of an analytical PAGE separation of RNase P M1 RNA processed, 5'-[^{32}P] radiolabeled library RNA. The label indicates whether RNase P M1 RNA was present (right) or not (left). **(C)** UV shadowing image of a denaturing 5% PAGE that was used to process library RNA (shown is the example of sub-round B in the first selection round). The strongest signal stems from RNase P M1 RNA due to its larger size and a 1.2-fold molar excess over the library RNA (see materials and methods). The 164 nucleotide long library was excised, eluted, and used for the selection step.

Figure S4

A

$$N(1E13) = 10^{13} * F_D^{N_{MutDes}} * (1 - (2.5 * F_D))^{(116 - 12 - N_{MutDes})}$$

B

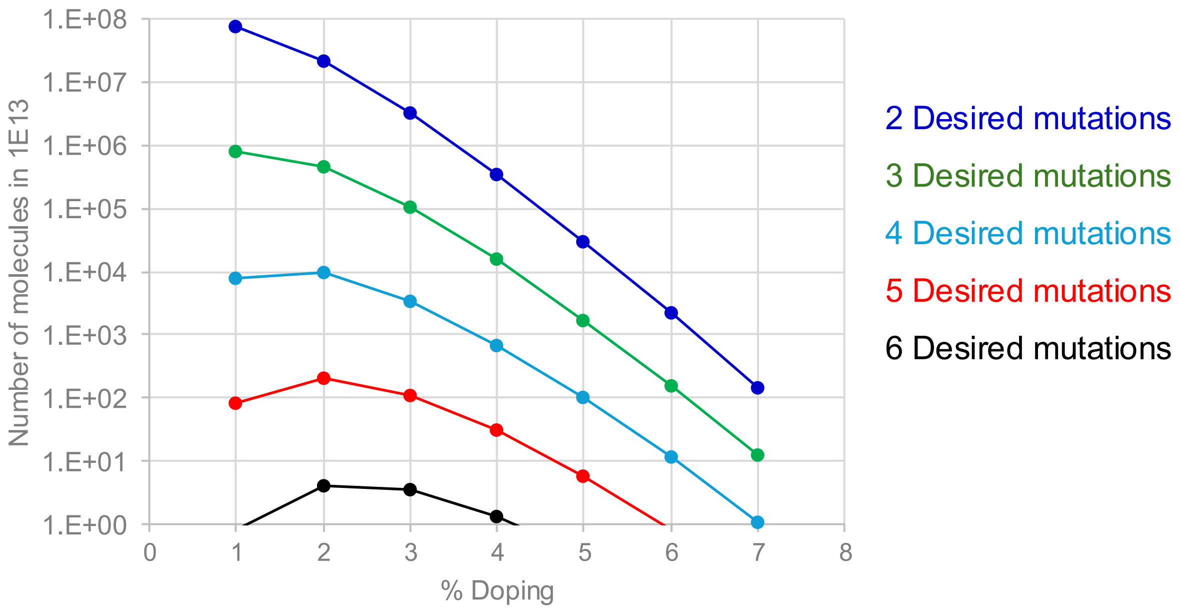

Figure S4. Expected coverage of mutations by the doped library. These deliberations were used to chose a promising doping ratio / mutagenesis rate for the doped library.

(A) The equation that was used to estimate the number of activity mediating variants in the doped library N(1E13) within 10^{13} different sequences as a function of doping ratio and the number of required / desired mutations. The doping fraction (F_D) is the fraction of molecules with a specific mutation. For example, if 94% of the molecules carry the wild-type sequence and 2% of molecules carry each of the three possible mutations then F_D=0.02. The number of desired mutations N_{MutDes} represents the number of desired mutations that need to appear within the same molecule, or cooperative mutations. The first factor of the equation is the total number of molecules(10^{13}), the second factor represents the frequency of molecules with the desired mutation, and the third factor estimates the frequency of molecules without deleterious mutations. This third factor assumes that every second AU pair can be replaced with GU, and every second GC pair can be replaced with GU, therefore the frequency of non-beneficial mutations is not based on the 3 possible mutations multiplied with the frequency of mutations but on 2.5 mutations multiplied with the frequency of mutaions ($2.5* F_D$). A second assumption is that not all non-beneficial mutations are deleterious but that 10% of positions in the ribozyme (12 positions in 116 doped positions) tolerate any mutation). Therefore the exponent of the third term ($116 - 12 - N_{MutDes}$) reduces the remaining number of doped positions ($116 - N_{MutDes}$) by a further 12.

(B) Graph showing the frequency of active library molecules as a function of doping ratio, and the specific number of desired mutations expected in 10^{13} different sequences of the library. The y-axis labels the number of molecules in the library of 10^{13} different sequences. Data are shown for 2 to 6 desired/ cooperative mutations, with the color coding shown on the right. Because it appears to contain the highest number of molecules with up to 5 or 6 cooperative mutations, a doping ratio of 2% was chosen.

Figure S5

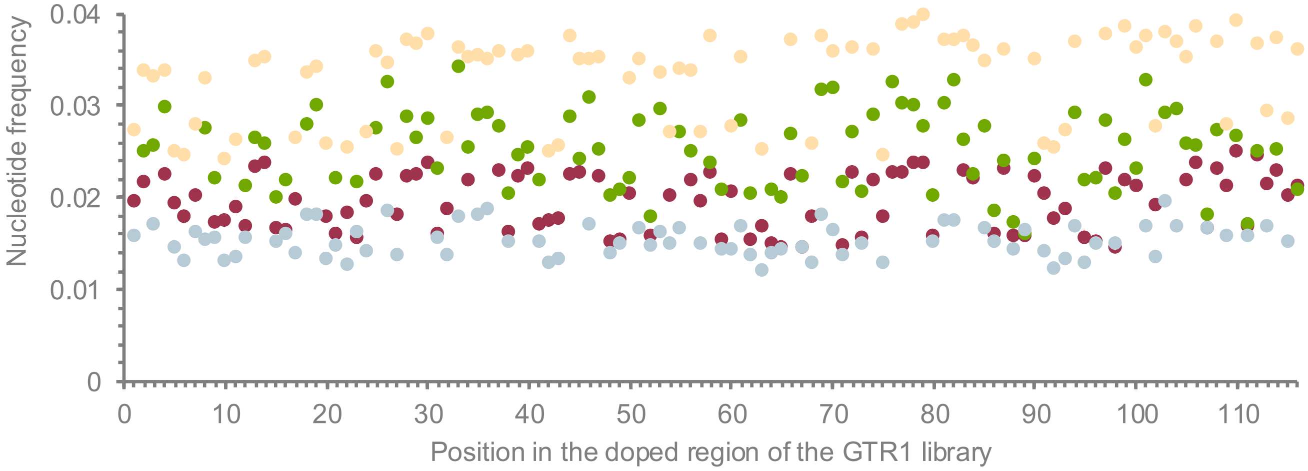

Figure S5. Frequency of mutations in the 116 nucleotide long doped region of the GTR1 doped library. The frequency was obtained from high throughput sequencing data of the library before selection. For each position the value is an average from more than 1 million sequences. While the desired ratio was 2% for all nucleotides and all positions (ordered as ultramer from IDT-DNA with hand-mixing), the average frequency was 3% for T (yellow), 2.5% for G (green), 2% (A) and 1.5% (C). These frequency values were used to calculate the enrichment of a specific mutation at a specific position.

Figure S6

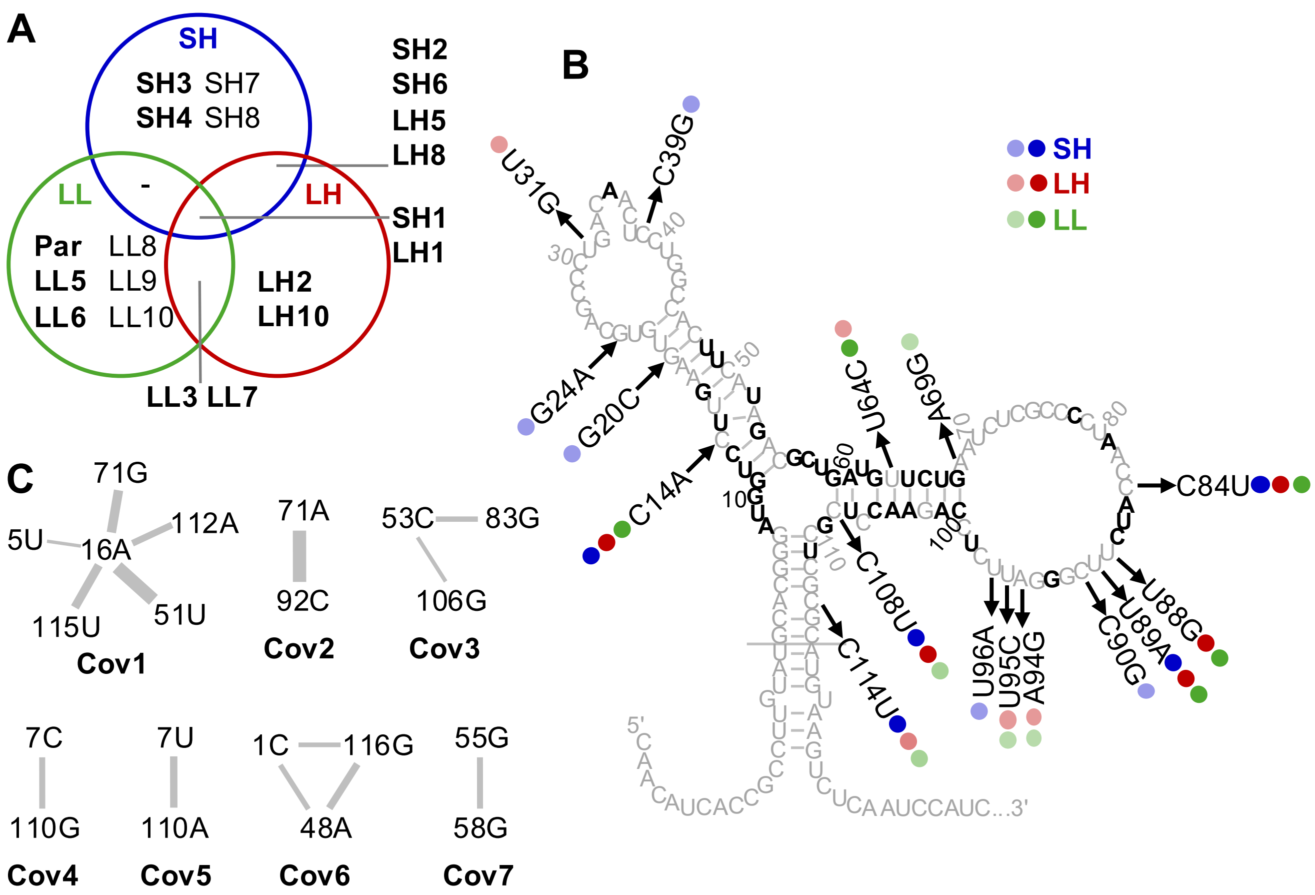

Figure S6. High Throughput Sequencing analysis of selected sequence populations. **(A)** Venn diagram of the top 20 sequences in the last round of selection for the three selection lines. The Sequence name indicates the selection line (SH = small droplets, high activity polymerase; LH = large droplets, high activity polymerase; LL = large droplets, low activity polymerase). The sequence name 'Par' indicates the 'parent' sequence, i.e. the sequence without any mutations, and the most frequent sequence in the initial doped library. The number in the sequence name shows the highest rank in the frequency of this sequence within any of the three selection lines. The numbers do not imply a quantitative comparison but a ranking order. Sequence names shown in bold were analyzed biochemically. **(B)** Positions of enriched mutations in the three separate lines of selection, projected on the published secondary structure model [1]. Letters in bold black illustrate nucleotides that were fully conserved within the top 20 sequences of the selection shown in (A). Arrows point to the most enriched mutations. Blue circles refer to mutations in selection line SH, red circles to selection line LH, and green circles to selection line LL. The five most enriched mutations in each line are shown in strong colors; the mutations ranked 6-10 in each line are shown semi-transparent. **(C)** Covariations between multiple mutations. The frequency of mutations occuring together more frequently than expected from their individual frequency was scored, and some of the most frequent covariations are shown here. The top 20 covariations from each of the three lines of evolution were considered. Covariations that conflicted with the SH4 sequence were not tested in the second round of activity analysis,which focused on variants of SH4. The thickness of the lines between the mutations describe the strength of the covariation.

Figure S7

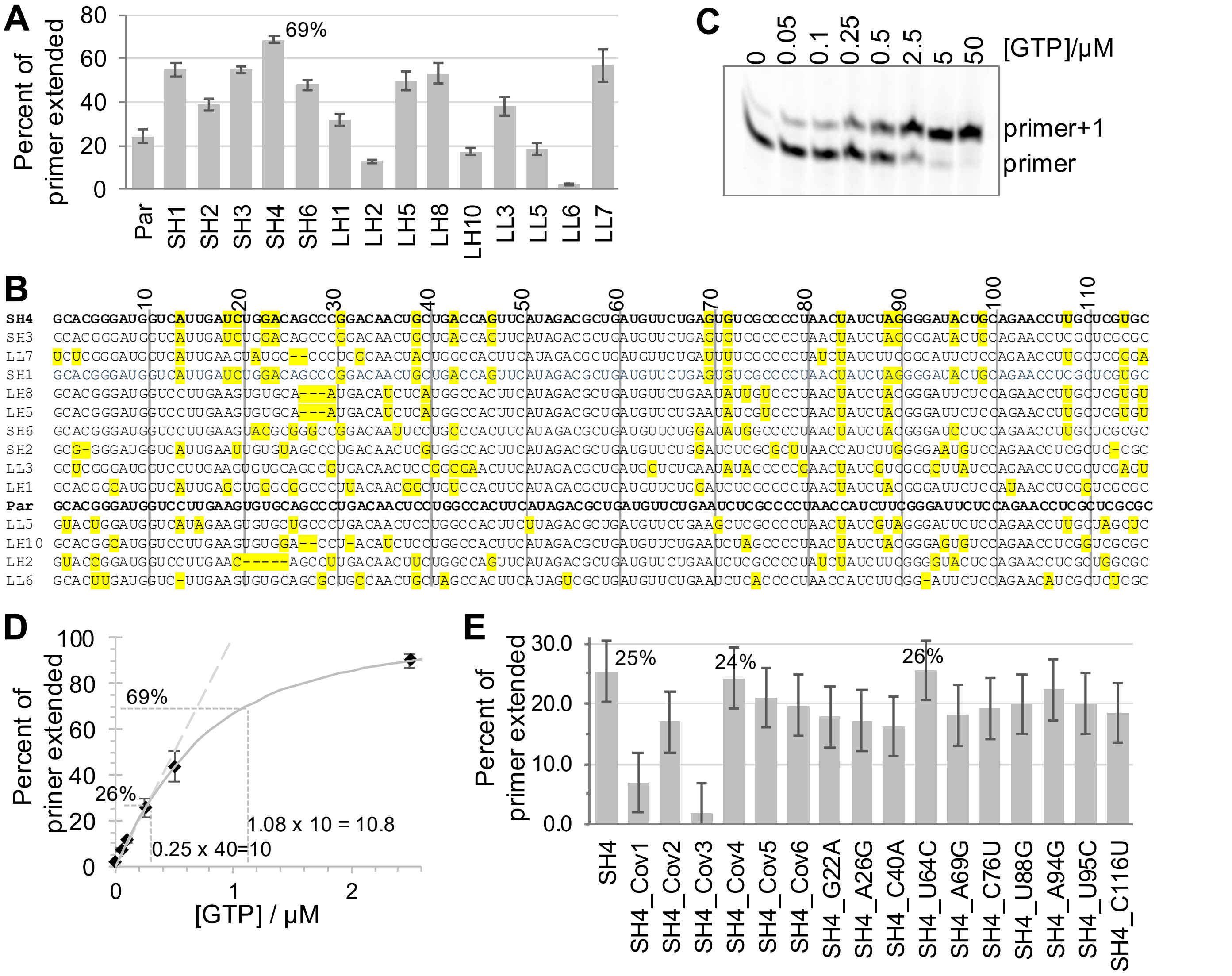

Figure S7. Biochemical identification of the most active GTP synthase variant. The assay used a polymerase ribozyme to extend a 10 nucleotide primer, using GTP that was generated by the GTP synthase ribozymes (Fig. 3A). All assays used 20 hour reaction times. **(A)** GTP synthase activity of 14 ribozyme variants from the selection lines SH, LL, and LH, as well as the parent ribozyme (Par). The strongest signal was obtained from the ribozyme variant SH4. This assay used a 10:1 ratio of primer to GTR1 variant, therefore it refers to the calibration curve in panel D that multiples with 10. **(B)** Sequences of the 15 biochemically tested sequences, sorted by their activity. The most active sequence SH4 and the parent sequence are labeled in bold. **(C)** Calibration of the GTP quantitation assay. Shown is a polyacrylamide gel image of reaction products, where labeled 10-nucleotide primers were extended by one nucleotide by the polymerase ribozyme, with increasing concentrations of GTP added. The reaction time was 20 hours. **(D)** Calibration curve to estimate the ribozyme's GTP turnover number (TON) from the percent of primers that were extended at different GTP concentrations. The assay was linear to about 40% of primer extended, as shown by the diagonal, dashed line that corresponds to a 100% use of GTP in the primer extension reaction. The gray curve is a double-exponential fit to the experimental data in (C). The horizontal and vertical dashed, gray lines show how the percent of primer extension was converted to the concentration of produced GTP. This GTP concentration was multiplied by 10 for the assay shown in (A), and multiplied by 40 for the data shown in (E) to account for the different dilution of the GTP synthesis reaction into the primer extension reaction (90-fold vs 360-fold, respectively). Error bars are standard deviations from three independent experiments. **(E)** GTP synthase activity of variants of the SH4 sequence. This assay used a 40-fold dilution of the GTP synthesis reaction to remain in the linear range of the assay, therefore it refers to the calibration curve in panel D that multiplies with 40. For each SH4 variant the mutation, or mutation covariations relative to SH4 are indicated (see figure S6 and text for details).

Figure S8

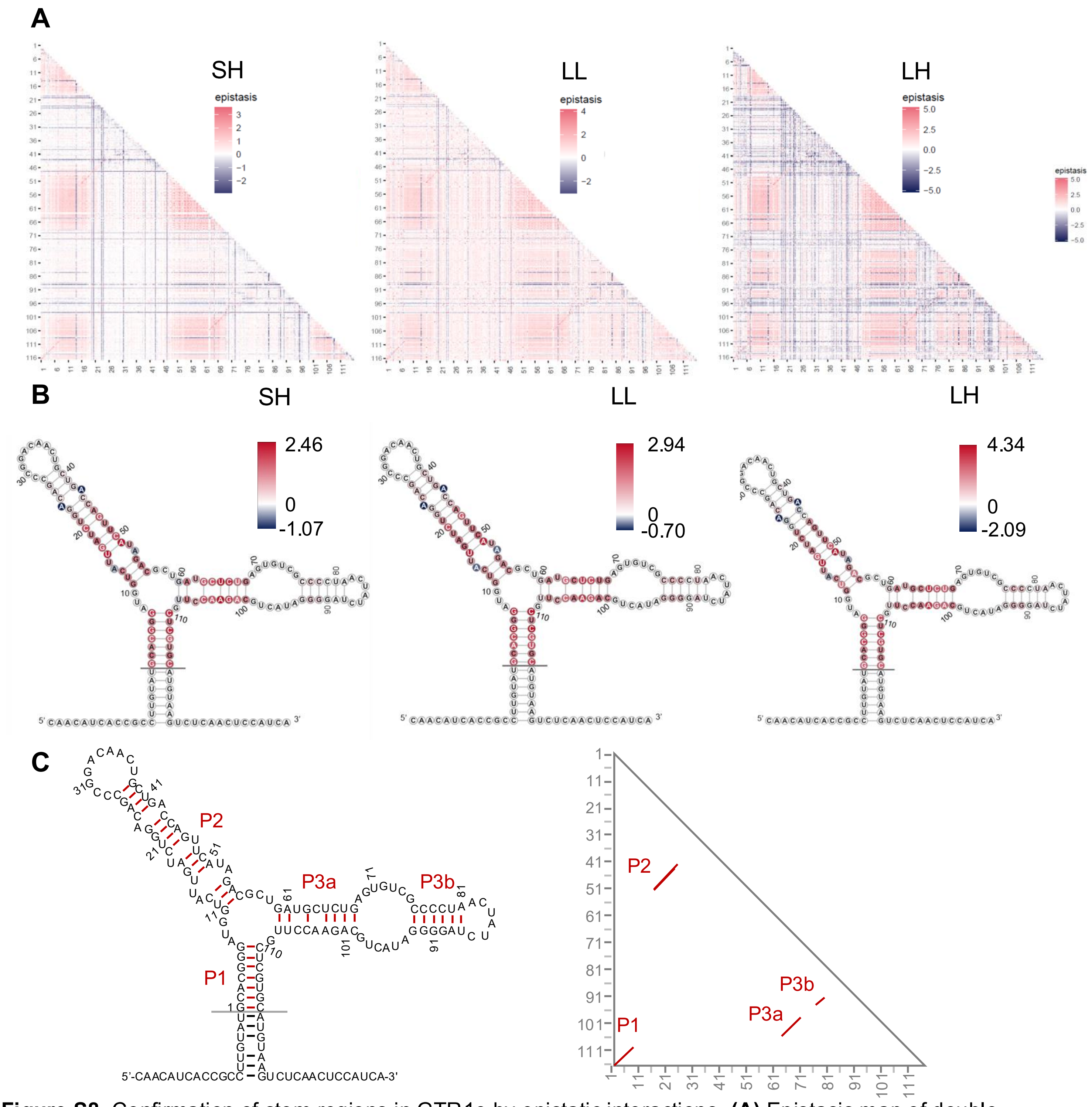

Figure S8. Confirmation of stem regions in GTR1e by epistatic interactions. **(A)** Epistasis map of double mutations. The plots show the epistasis of every two mutations in the last selection round of line SH (left), LL (center) and LH (right), respectively. The selection line is indicated for each plot. Values larger than parent sequences (>0) are colored in red, while values lower than parent sequences (<0) are in blue. The axes show all mutations at each position, in the order of A, G, C, T. **(B)** Secondary structure of GTR1e with the mean epistasis of basepair-preserving mutations in the stem regions mapped onto them. The selection lines SH (left), LL (center) and LH (right) are indicated. Only four stem regions were considered (1-7: 116-110; 11-27: 56-40; 60-68:108-100; 76-81: 93-88). Epistasis higher than parent sequences (>0) were colored in red while the epistasis lower than parent sequences (<0) were colored in blue. The plots were generated with the software cytoscape. **(C)** Schematic that illustrates the connection between secondary structure and epistatic interaction maps. The secondary structure (left) is annotated with the stems P1-P3 (red) in the doped region (above the gray line). The schematic on the right shows where the stems (red lines; annotated as P1-P3) are represented in the epistatic interaction maps.

Figure S9

A

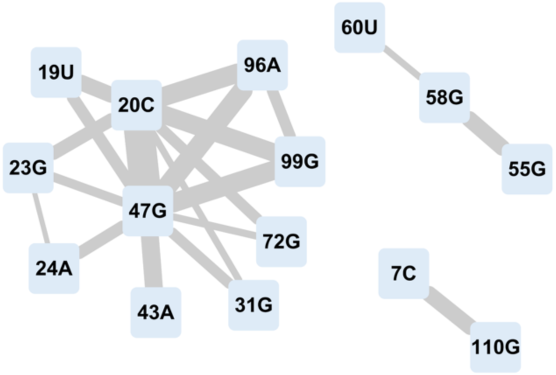

B

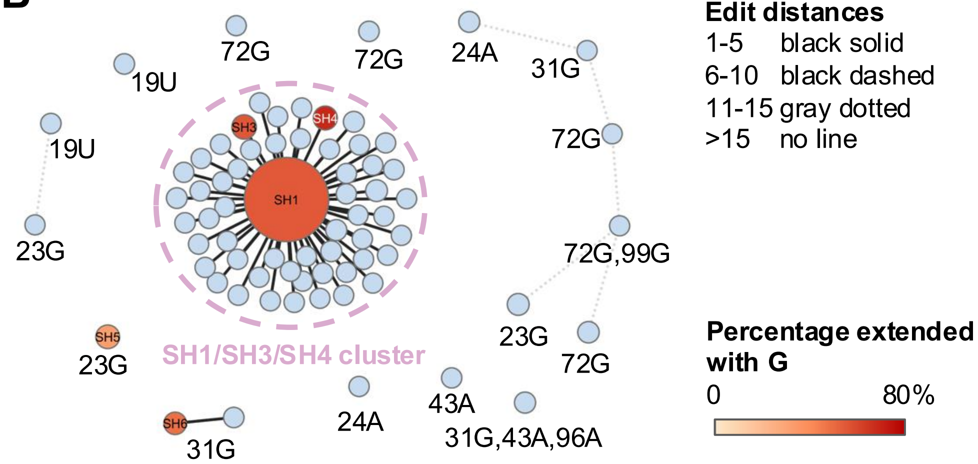

Figure S9. Network that showed up as false positive in epistatic interaction analysis. **(A)** The top 20 epistatic interactions of the SH line round 3 library. The thickness of lines is linearly correlated to the epitasis value between the two connected mutations. The plots were generated by the software cytoscape. **(B)** Edit distances between sequences show the set of ten mutations in (A). Increasing distances are represented as black solid lines (1-5 edits), black dashed lines (6-10 edits), gray dotted lines (11-15 edits) and the absence of lines (more than 15 edits). Each bubble corresponds to one sequence. Only sequences are represented that contain at least one of the ten mutations, with a read number of at least 34 RPM (reads per million). These ten mutations are conserved between the three highly active sequences SH1, SH3, and SH4 (red bubbles), which all exist within the same cluster (pink dashed circle).

Five of the shown sequences were tested biochemically; the activity of these five sequences was measured as the percentage of primer extended by G. Their activity is represented as shades of yellow and red as given by the scale. All other sequences are represented by light blue bubbles.

The separation between this cluster and other sequences, and the absence of the same ten mutations in other clusters or sequences show that the conservation of these ten mutations is *not a valid signal for epistatic interactions because all sequences within the cluster originated from the same founder sequence*.

Figure S10

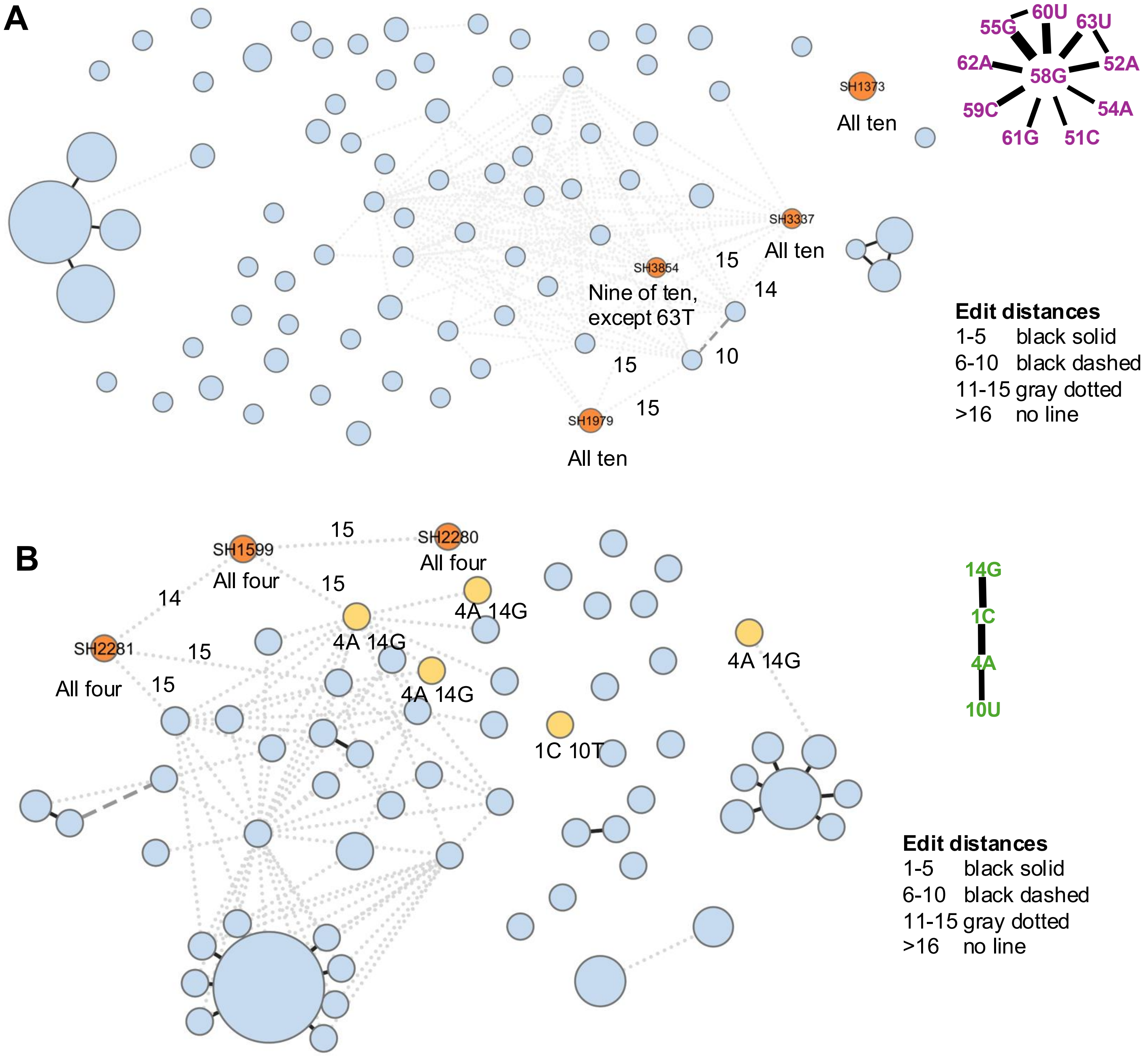

Figure S10. Apparently valid epistatic interaction networks shown in figure 2. Both networks remained in the top 20 epistatic interactions, after the ~6,000 sequences of the SH4 cluster were removed. Mutations at these positions were low in abundance and therefore most clusters only had one representative sequence. The separation between these sequences were more than 14 edit distance and therefore most likely to have originated from different founder sequences. The existence of these sequences showed that about three sequences containing the ten mutation network and about three sequences containing the four mutation network originated from seperate founder sequences. The plots were generated by the software cytoscape. **(A)** Network of 10 mutations (51C, 52A, 54A, 55G, 58G, 59C, 60U, 61G, 62A, 63U). The corresponding schematic of figure 2 is shown on the right, with the thickness of lines being linearly correlated to the epitasis value between the two connected mutations. Three clusters showed all ten mutations, and one cluster showed nine of the ten mutations as indicated. Unlabeled sequences show less than nine mutations. The closest edit distances are labeled with the number of edits. **(B)** Network of 4 mutations (1C, 4A, 10U, 14G). The corresponding schematic of figure 2 is shown on the right, with the thickness of lines being linearly correlated to the epitasis value between the two connected mutations. Three sequences contained all four mutations and five sequences contained two of the four mutations. All other bubbles represent sequences with only one of the four mutations.

Figure S11

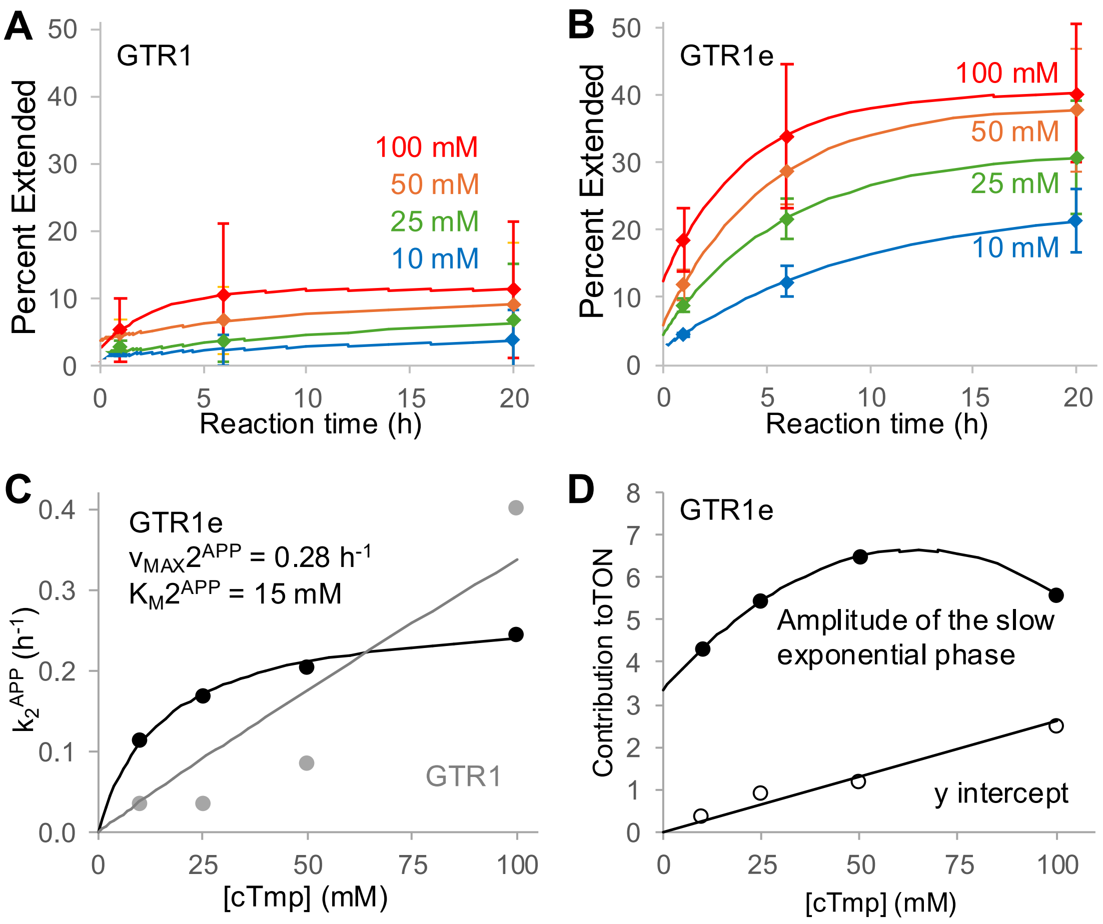

Figure S11. Dependence of Ribozyme-catalyzed guanosine triphosphorylation on the concentration of cTmp. In all reactions, the concentrations of RNAs in the coupled reaction were set such a fraction of 0.5 for the extended primer correlated with a turnover number of 10. **(A)** Reaction kinetics of the ribozyme GTR1 in the presence of increasing concentrations of cTmp. The lines were fitted to the data using the Michaelis-Mented equation with an added y-intercept. The concentrations of cTmp are labeled by color coding. Error bars denote standard deviations of triplicate experiments. **(B)** As in (A) but for for GTR1e. **(C)** Plot of the initial slopes of the fitted equations in (A) and (B), describing the dependence of the rate of the slow exponential phase (k_2^{APP}) on cTmp concentration. For GTR1e (black), the apparent maximal rate and K_M^{APP} are given. For GTR1 (gray), the data did not allow determinig meaningful constants. **(D)** Plot of the amplitudes of the fast phase (empty circles) and the slow phase (filled circles) as a function of cTmp concentration. The fast phase (y intercept; empty circles) followed a linear relationship with cTmp concentration (y = 0.026 * [cTmp] / mM). The parabolic curve fit for the amplitude of the slow, exponential function (filled circles) was maximal at 64 mM cTmp, where it contributed 6.7 to the total TON.

Figure S12

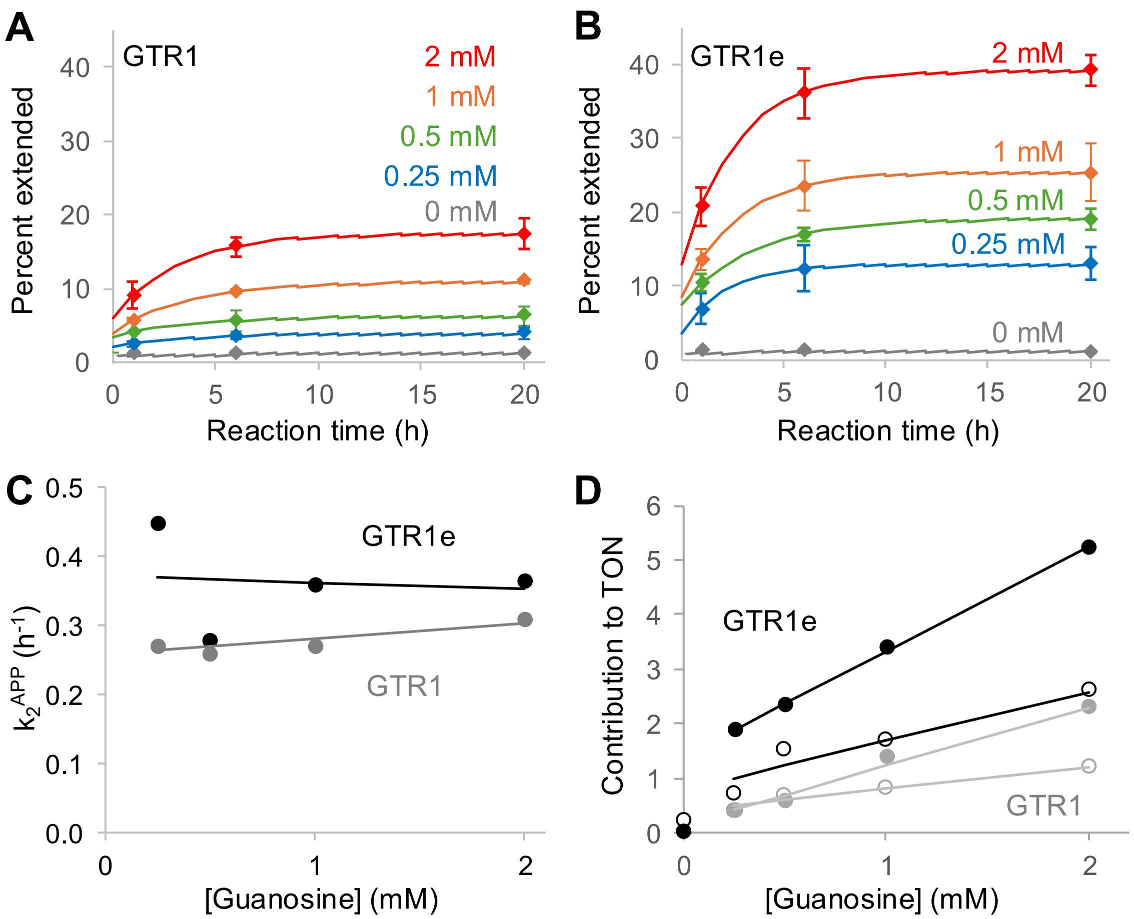

Figure S12. Dependence of ribozyme catalyzed GTP synthesis on guanosine concentration. **(A)** Plot for GTR1, of the fraction of extended primer as function of the reaction time at different concentrations of guanosine at 2 mM (red), 1 mM (orange), 0.5 mM cTmp (green), and 10 mM (blue). The lines are single-exponential functions fitted with a y-offset, corresponding to a fast initial burst of a biphasic fit. **(B)** As in (A) but for GTR1e. **(C)** Plot of the average reaction rates of GTR1e (black) and GTR1 (gray) that were determined in (A) and (B) from the slow single-exponential rates. These slow rates do not include the y-offset (initial burst phase). The rates for both ribozymes appeared independent of the guanosine concentration in the measured range, as shown by the approximately horizontal linear fit to the data (black and gray lines). **(D)** Dependence of the amplitudes of y intercepts and slow phase on the guanosine concentration. Symbols denote the y intercept for GTR1e (empty black circles), the slow phase for GTR1e (filled black circles), the y intercept for GTR1 (empty gray circles) and the slow phase for GTR1 (filled gray circles). All data were described well with linear fits between 0.25 mM and 2 mM guanosine (black and gray lines), suggesting that for both ribozymes the TON can be further increased with tighter guanosine binding.

References:

[1] Akoopie et al. (2021) *Sci. Adv.* 7 (41) eabj7487
[2] Akoopie & Müller (2018) *Nucl. Acids Res.* 46 (20) 10589
[3] Magde et al. (2021) *ACS Omega* 6 (33) 21773
[4] Higgs & Müller (2025) J. R.Soc. Interface 22 (225) 20240878
[5] McRae et al. (2024) PNAS 121 (3) e2313332121
